# Oxygen Reduction Reaction Catalyzed by Carbon-Supported Platinum Few-Atom Clusters: Significant Enhancement by Doping of Atomic Cobalt

**DOI:** 10.34133/2020/9167829

**Published:** 2020-11-06

**Authors:** Bingzhang Lu, Qiming Liu, Forrest Nichols, Rene Mercado, David Morris, Ning Li, Peng Zhang, Peng Gao, Yuan Ping, Shaowei Chen

**Affiliations:** ^1^Department of Chemistry and Biochemistry, University of California, 1156 High Street, Santa Cruz, California 950564, USA; ^2^Department of Chemistry, Dalhousie University, 6274 Coburg Road, Halifax, Nova Scotia, Canada B3H 4R2; ^3^International Center for Quantum Materials, School of Physics, Peking University, Beijing 100871, China; ^4^Electron Microscopy Laboratory, School of Physics, Peking University, Beijing 100871, China; ^5^Collaborative Innovation Centre of Quantum Matter, Beijing 100871, China

## Abstract

Oxygen reduction reaction (ORR) plays an important role in dictating the performance of various electrochemical energy technologies. As platinum nanoparticles have served as the catalysts of choice towards ORR, minimizing the cost of the catalysts by diminishing the platinum nanoparticle size has become a critical route to advancing the technological development. Herein, first-principle calculations show that carbon-supported Pt_9_ clusters represent the threshold domain size, and the ORR activity can be significantly improved by doping of adjacent cobalt atoms. This is confirmed experimentally, where platinum and cobalt are dispersed in nitrogen-doped carbon nanowires in varied forms, single atoms, few-atom clusters, and nanoparticles, depending on the initial feeds. The sample consisting primarily of Pt_2~7_ clusters doped with atomic Co species exhibits the best mass activity among the series, with a current density of 4.16 A mg_Pt_^−1^ at +0.85 V vs. RHE that is almost 50 times higher than that of commercial Pt/C.

## 1. Introduction

Oxygen reduction reaction (ORR) is an important process for a range of electrochemical energy technologies, such as fuel cells and metal-air batteries [1–3]. Currently, Pt nanoparticles (Pt NPs) are the catalysts of choice towards ORR (state-of-the-art Pt/C catalysts ca. 3 nm in diameter [4]), which account for over 50% of the total device cost and significantly hamper the widespread commercialization of the technologies [5]. There are two leading strategies to reduce the catalyst cost. One is to develop platinum-free alternatives, such as heteroatom-doped carbon nanocomposites [6–9]. Yet, despite substantial progress in the alkaline media, it remains challenging to achieve a viable activity in acid, as compared to Pt/C. The other is to reduce the amount of Pt used and hence to diminish the catalyst cost but without compromising the electrocatalytic performance [10–14]. One intuitive strategy is to reduce the size of Pt NPs by taking advantage of the increasing surface-to-volume ratio and hence enhanced utilization of the Pt atoms. For instance, Pt_12_ clusters (dia. 0.9 nm) have been found to outperform Pt_28_ (1.0 nm) and Pt_60_ clusters (1.1 nm) towards ORR, with a mass activity one order of magnitude higher than that of commercial Pt/C [15]. One may argue then that Pt single-atom catalysts (SACs) would be the ultimate solution, thanks to the maximal atomic utilization [16]. Yet, Pt SACs exhibit mostly a limited ORR activity, whereby O_2_ undergoes 2e^−^ reduction to H_2_O_2_, rather than the 4e^−^ pathway to H_2_O [17, 18]. This has been observed with Pt SACs supported on a wide range of substrate matrices, such as TiC/TiN [19, 20], reduced graphene oxide [17], S-doped carbon [18], and N-doped carbon [21]. The limited ORR performance of Pt SACs, in comparison to that of Pt NPs, is primarily accounted for by the isolated binding sites that render it energetically difficult to adsorb oxygen and to break the O-O bond [16, 22]. However, the mechanistic details remain under active debates, and the threshold of the Pt domain size for ORR has been largely unexplored.

Note that for conventional Pt NP catalysts, alloying with a second, nonnoble metal is an effective strategy to enhance the ORR activity, primarily due to electronic and geometric effects [23–27]. For instance, Stamenkovic et al. [28] observed that 3d transition metals can shift the d-band center of Pt and manipulate the ORR activity. Strasser et al. [29] discovered that lattice strain of Pt alloys can significantly weaken oxygen adsorption and enhance the ORR activity. Note that alloying also leads to a reduced loading of Pt in the nanoparticle catalysts, a key factor to enhance the mass activity [30]. For instance, Oezaslan and Strasser [31] observed a significant improvement of the ORR mass activity with PtCo, as compared to Pt. In another study, Chong et al. [32] synthesized a Pt_3_Co alloy NP (dia. 5.6 nm) catalyst with an ultralow Pt loading of 2.7 wt%, which exhibited a mass activity several hundred times better than that of commercial 20 wt% Pt/C, due to the synergistic interaction between Pt_3_Co NP and CoN_4_ within the carbon matrix. Yet, to the best of our knowledge, there have been no studies of alloying at the Pt few-atom or single-atom level thus far. This is the primary motivation of the present study.

Herein, we first carry out first-principle calculations and examine and compared the ORR activities of various Pt sites in a range of domain size. The results show only limited ORR activity with subnanometer-sized Pt clusters and Pt SACs, as compared to Pt slab, primarily due to their inability to adsorb oxygen. Yet the addition of neighboring Co atoms can significantly boost the electrocatalytic performance. These theoretical findings are indeed confirmed in experimental measurements with two sets of nanocomposite samples, Pt and Pt/Co few-atom clusters supported on nitrogen-doped carbon. Among the series, Pt_2~7_ clusters doped with Co atomic species stand out as the optimal catalyst with a mass activity 48 times that of commercial Pt/C. This is ascribed to the synergistic interactions between Pt and adjacent Co.

## 2. Results and Discussion

### 2.1. First-Principle Calculations

ORR is a multiple-electron reaction process, typically involving the following steps [33–35]:

(1) $$\begin{array}{c} {}*+{\text{O}}_{2}+{\text{H}}^{+}+{\text{e}}^{-}\to {\text{OOH}}^{*}\;\Delta G_{1} \end{array}$$

(2) $$\begin{array}{c} {\text{OOH}}^{*}+{\text{H}}^{+}+{\text{e}}^{-}\to {\text{O}}^{*}+{\text{H}}_{2}\text{O}\;\Delta G_{2} \end{array}$$

(3) $$\begin{array}{c} {\text{O}}^{*}+{\text{H}}^{+}+{\text{e}}^{-}\to {\text{OH}}^{*}\;\Delta G_{3} \end{array}$$

(4) $$\begin{array}{c} {\text{OH}}^{*}+{\text{H}}^{+}+{\text{e}}^{-}\to {\text{H}}_{2}\text{O}+*\;\Delta G_{4} \end{array}$$

where ∗ represents the active site.

**Figure 1:**
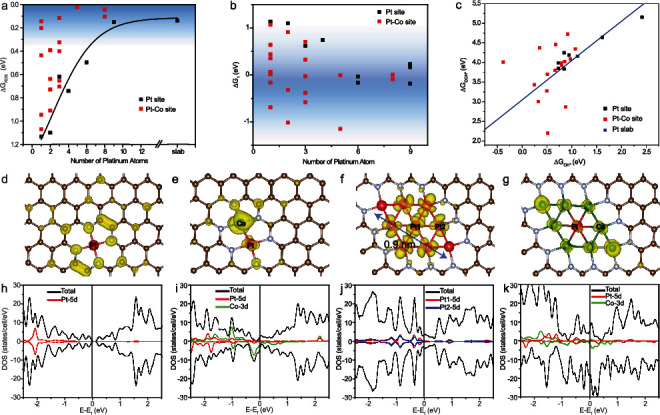
Results of DFT calculations of Pt and Pt-Co clusters of different sizes embedded in a nitrogen-doped carbon matrix. The potential is set to +0.90 V vs. RHE. (a) Gibbs free energy of the rate determine step (Δ*G*_RDS_) versus platinum clusters of different sizes (Pt_*x*_). (b) Gibbs free energy of the first-electron reduction (Δ*G*_1_) versus platinum clusters of different sizes (Pt_*x*_). The dark blue regions in (a) and (b) indicate the range of optimal energy for ORR. (c) Correlation between adsorption free energy of OOH∗ and OH∗ intermediates on Pt sites on carbon (black squares), Pt-Co sites in carbon (red squares), and Pt slabs (blue line). (d–g) Wave function module square of selected configurations of Pt and Pt-Co in carbon near the Fermi level with an isosurface value of 0.001 e/au^3^. (h–k) The corresponding density of states (DOS) of configurations (d–g). The Pt, Co, N, and C atoms are denoted by red, green, grey, and brown balls, respectively.

The ORR activity has been argued to be most likely limited by two steps, the first-electron reduction of oxygen (Equation (1)) and reduction of adsorbed hydroxy to water and its desorption from the catalyst surface (Equation (4)).  Figure 1(a) depicts the calculated Gibbs free energy of the rate determine step (Δ*G*_RDS_, which is defined as the highest of the four Δ*G*'s, Table S1) in ORR, catalyzed by Pt clusters of different sizes (Pt_*x*_, *x* = 1 to 9, black squares), and supported on nitrogen-doped carbon, in comparison to that of a Pt slab (Figure S1). One can see that Δ*G*_RDS_ decreases monotonically with increasing Pt domain size and becomes largely leveled off at Pt_9_ (from 1.13 eV for Pt_1_ to 0.17 eV for Pt_9_, as compared to 0.14 eV for Pt slab) (Figure S1 and Note S1), indicating that large NPs, rather than SACs, are preferred for ORR. This suggests that the domain size of Pt should be at least 9 atoms (ca. 0.9 nm in diameter) for optimal ORR activity. Interestingly, incorporation of an adjacent Co atom forming a Pt-Co pair significantly enhances the ORR activity. The red squares in Figure 1(a) are the Δ*G*_RDS_ of a range of configurations containing a varied number of Co and Pt (Figure S1–S3). One can see that these PtCo sites all exhibit a markedly lower Δ*G*_RDS_ than the corresponding Pt counterparts, suggesting that the ORR activity of Pt clusters can be readily activated by neighboring Co, and the enhancement becomes drastically intensified with an increasing number of the Pt-Co pairs (Note S2).

Figure 1(b) depicts the corresponding Gibbs free energy of the first-electron reduction of oxygen (Δ*G*_1_, Equation (1)). One can see that Δ*G*_1_ is positive for small Pt domains (*x* < 6, black squares), far above the optimal energy of ca. -0.3 eV to 0.0 eV [36] that is highlighted in dark blue, signifying energetically unfavorable adsorption of oxygen. Yet, at any Pt domain size, the incorporation of adjacent cobalt atoms leads to a marked shift of Δ*G*_1_, some very close to the blue region and some others even below. This is likely because of the formation of Pt-Co bonding pairs that facilitate bridge-adsorption of oxygen, which decreases the reaction energy (Figure S4), and some configurations are even so oxytropic that they can spontaneously break the O-O bond during this first-electron reduction of oxygen (e.g., Pt_3_Co_3_, Figure S1), leading to a negative Δ*G*_1_. Moreover, based on the adsorption energy of the OOH∗ and OH∗ intermediates, small Pt-Co clusters can be found to deviate from the linear relationship, as shown in Figure 1(c), where the energies of a number of configurations (red squares) are randomly scattered away from the line calculated for the Pt slab. Note that the linear relationship has been observed extensively in prior studies with large Pt NPs [33, 34, 37]. Breaking of the linear relationship has been reported with Pt SACs, which show unexpected ORR activity [38].

To better understand the role of cobalt and platinum in these bimetallic catalyst configurations, from the viewpoints of electronic structure and chemical bonding, the projected density of states (DOS) near the Fermi level was then calculated for several typical candidate structures (Figures 1(d)–1(g)). For isolated Pt_1_ embedded within N-doped carbon, the wave function near the Fermi level has no contribution from the Pt atom, but mainly resides at the neighboring N and C sites instead (yellow regions in Figure 1(d)); moreover, the DOS is low near the Fermi level, with almost no contribution from the Pt 5d orbital (Figure 1(h)). This indicates that isolated Pt_1_ have no activated electrons to participate in reactions, in agreement with the low ORR activity observed experimentally. By contrast, for the dimeric Pt_1_Co_1_ pair (Figures 1(e) and 1(i)), there is an apparent contribution of the Co 3d orbital to the DOS and wave function near the Fermi level, and an obvious overlap between the Pt 5d and Co 3d orbitals, making the Pt site much more favorable for oxygen adsorption than Pt single atom alone. Two other extreme cases with six coordinated Pt should also be noticed. For a Pt_9_ cluster supported in N-doped carbon (Figures 1(f) and 1(j)), the activity of the central and edge Pt atoms is better than or very close to that of the platinum slab (Δ*G*_RDS_ = 0.06 eV and 0.17 eV, respectively, *vide ante*). The wave function near the Fermi level is distributed over the Pt atoms, and the Pt 5d orbital contribution to the DOS near the Fermi level leads to favorable oxygen adsorption. Pt_1_Co_8_ is another case (Δ*G*_RDS_ = 0.15 eV) with six co-coordinated Pt. Both the platinum and cobalt atoms contribute to the large DOS near the Fermi level, which can be also seen in the wave function that is distributed over Pt and Co at the Fermi level, further enhancing platinum's ability for oxygen adsorption as well as improving electrical conductivity (Figures 1(g) and 1(k)).

Bader charge is adopted to analyze the charge distribution on the individual atoms within the system. One can see a significant charge gain on the Pt atom as a result of Pt-Co direct bonding interactions. As shown in Figure S5 and Table S2, for the Pt-Co pair, the Pt charge has ca. 0.4 e atom^−1^ more than that of an isolated Pt atom or a Pt atom not directly bonded with Co (PtCo∗ in Figure S5). This further explains that the direct bonding between Co and Pt improves the activity much more than the nondirect bonding case, as discussed earlier. The charge on the Co atom does not have a significant difference, as compared to isolated Co atoms in carbon. This charge gain of Pt is also confirmed by X-ray absorption spectroscopy (XAS) and X-ray photoelectron spectroscopy (XPS) measurements (vide infra).

Taken together, results from these calculations suggest that (a) in order to attain a reasonable ORR activity, the Pt domain must reach a minimum size (e.g., *x* ≥ 9), and (b) neighboring Co can substantially influence the electronic structure of the Pt atoms and facilitate oxygen adsorption, but only when Pt and Co are directly bonded. Within this context, two sets of catalysts based on carbon-supported Pt and PtCo clusters are synthesized, where the activity trends are indeed consistent with the theoretical prediction. From these, the platinum mass activity for ORR is maximized.

### 2.2. Sample Synthesis and Structural Characterization

**Scheme 1:**
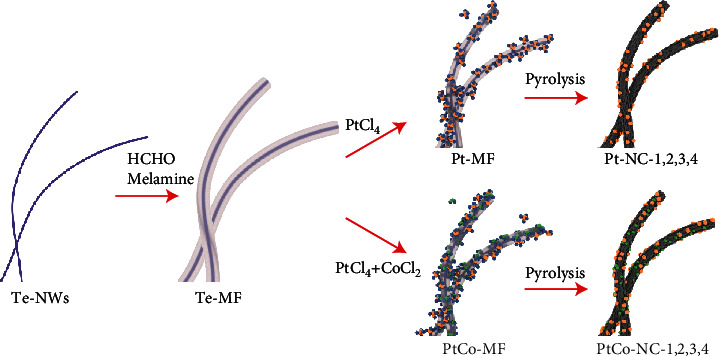
Schematic of the preparation of the Pt-NC and PtCo-NC samples.

The preparation of the samples is schematically shown in Scheme 1. Tellurium nanowires (Te NWs) were used as thermally removable templates and coated with a melamine-formaldehyde (MF) resin shell that served as the carbon and nitrogen sources [39–41]. A different amount of PtCl_4_ (and CoCl_2_) was then added to the resulting core@shell composite (Te-MF), and subsequent pyrolysis of the mixtures at 900°C led to the formation of nitrogen-doped carbon nanowire-supported Pt (PtCo) [36, 42]. Four samples of each series were prepared, which were denoted as Pt-NC-1, Pt-NC-2, Pt-NC-3, and Pt-NC-4 for the samples containing only Pt, and PtCo-NC-1, PtCo-NC-2, PtCo-NC-3, and PtCo-NC-4 for those containing both Pt and Co. The concentration of the PtCl_4_ precursor was kept the same for samples with the sample number (e.g., Pt-NC-1 and PtCo-NC-1 were prepared at the same PtCl_4_ concentration). Inductively coupled plasma-optical emission spectrometric (ICP-OES) measurements show that the Pt contents in the final samples were also close, ca. 0.25 wt% for both Pt-NC-1 and PtCo-NC-1, 0.41 wt% for Pt-NC-2 and 0.50 wt% for PtCo-NC-2, 1.39 wt% for Pt-NC-3 and 0.95 wt% for PtCo-NC-3, and 4.75 wt% for Pt-NC-4 and 6.29 wt% for PtCo-NC-4 (Table S3). Note that these are all significantly lower than that in state-of-the art Pt/C (20 wt%).

**Figure 2:**
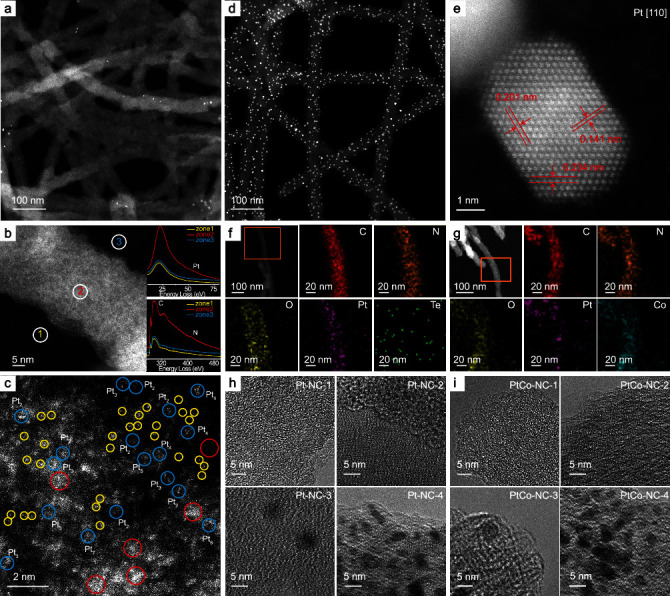
TEM studies of Pt-NC and PtCo-NC nanowires. (a–c) HAADF-STEM images of Pt-NC-3. Insets of (b) are the EELS spectra at three selected areas. In (c), yellow circles signify Pt single atoms, blue circles highlight few-atom Pt_2~7_ clusters, and red circles denote Pt_9_ or larger clusters. (d) HAADF-STEM image of Pt-NC-4. (e) High-resolution TEM image of a Pt NP of Pt-NC-4. EDS elemental mapping studies of (f) Pt-NC-3 and (g) PtCo-NC-3. High-resolution TEM images of the (h) Pt-NC and (i) PtCo-NC samples.

The obtained samples were first characterized by transmission electron microscopic (TEM) measurements (Figure 2). Figure 2(a) shows a representative TEM image of Pt-NC-3. One can see that the nanowires have a diameter of ca. 40 nm and length of several microns, with only a handful of NPs, suggesting that Pt was mostly in the forms of few-atom clusters and single atoms. Figure 2(b) shows the corresponding elemental mapping analyses based on electron energy loss spectroscopic (EELS) measurements. It can be seen that the signals of the Pt O_2,3_-edge (ca. 50 eV), C K-edge (ca. 284 eV), and N K-edge (ca. 401 eV) are all prominently higher in the nanowires (zone 2, red curves) than in the background (zone 1, yellow curves; and zone 3, blue curves), indicating that Pt was embedded in the nitrogen-doped carbon structure. Figure 2(c) is a high-angle annular dark-field scanning transmission electron microscopy (HAADF-STEM) image of Pt-NC-3, where isolated Pt atoms (yellow circles) can be found throughout the carbon matrix, along with a number of few-atom clusters (Pt_2~7_, blue circles) and a few larger clusters (Pt_≥9_, red circles), which are good representations of the calculation models (Pt_*x*_) in Figure S1.

Notably, for samples prepared at lower Pt loadings (i.e., Pt-NC-1 and Pt-NC-2), no Pt NPs can be found (Figure 2(h) and Figure S6), suggesting that Pt is mostly dispersed within the carbon matrix as few-atom clusters and/or isolated atoms. Yet for Pt-NC-4, which was prepared with a PtCl_4_ concentration 5 times that for Pt-NC-3, one can see that a number of Pt NPs (dia. 5 nm) were formed on the carbon nanowires (Figures 2(d) and 2(e)), which exhibit clearly defined lattice fringes with the interplanar spacings of 0.141, 0.201, and 0.234 nm due to the (110), (001), and (111) vectors of the {110} facet of *fcc* Pt (card 00-001-1190) [43].

It is worth noting that the addition of Co did not lead to an apparent variation of the overall sample morphology. This is manifested in elemental mapping analysis of the Pt-NC-3 (Figure 2(f)) and PtCo-NC-3 (Figure 2(g)) samples, where the elements of C, N, O, and Pt can all be readily identified and dispersed in the nanowires (Co in PtCo-NC-3). Similar to the Pt-NC series, for PtCo-NC-1 and PtCo-NC-2 (Figure 2(i) and Figure S7), the metal species are dispersed within the carbon skeletons as isolated atoms and/or few-atom clusters, and NPs started to emerge in PtCo-NC-3 and became prominent in PtCo-NC-4 (at even higher metal loadings, NPs became the dominant species in the samples, Figure S8).

**Figure 3:**
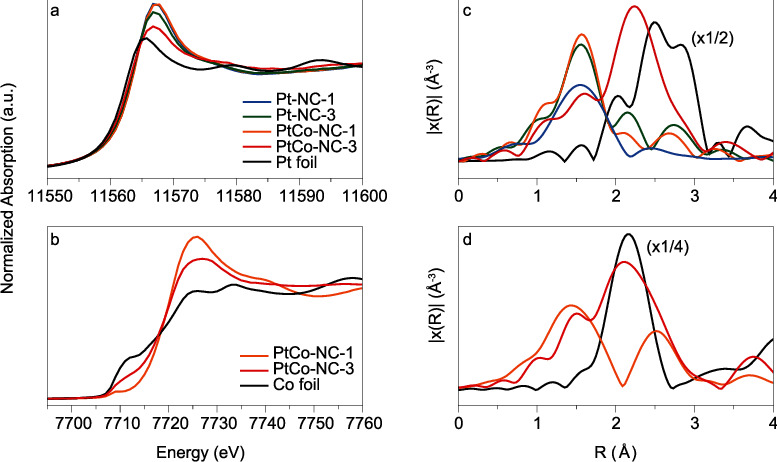
Spectroscopic characterization of the Pt-NC and PtCo-NC samples. (a) Pt XANES of Pt-NC-1, Pt-NC-3, PtCo-NC-1, PtCo-NC-3, and Pt coil. (b) Co XANES spectra of PtCo-NC-1, PtCo-NC-3, and Co coil. (c) Pt FT-EXAFS spectra of Pt-NC-1, Pt-NC-3, PtCo-NC-1, PtCo-NC-3, and Pt coil. (d) Co FT-EXAFS spectra of PtCo-NC-1, PtCo-NC-3, and Co coil.

The atomic configurations of the metal centers were then examined by XAS measurements.  Figure 3(a) shows the Pt L_3_-edge X-ray absorption near-edge structure (XANES) spectra of Pt-NC-1, Pt-NC-3, PtCo-NC-1, PtCo-NC-3, and Pt foil. Pt-NC-1 and PtCo-NC-1 can be seen to show a stronger white line (i.e., the first peak following the absorption edge) than the others, indicating a higher oxidation state of Pt, which is consistent with that of Pt single atoms in a carbon matrix [44–46]. For Pt-NC-3 and PtCo-NC-3, the absorption edge is higher than that of the Pt foil, but lower than those of Pt-NC-1 and PtCo-NC-1. This can be accounted for by the partial clustering of Pt and Co, which led to a reduced Pt oxidation state, as compared to that in Pt-NC-1 and PtCo-NC-1. Notably, the white line of Pt-NC-3 is somewhat stronger than that of PtCo-NC-3, likely due to electron transfer from Co to Pt in the latter. The corresponding XANES spectra of the Co K-edge are shown in Figure 3(b). Consistently, PtCo-NC-1 and PtCo-NC-3 exhibit a stronger white line at Co K-edge than the Co foil, and the pre-edge peak disappears, where the Co oxidation state is consistent with the formation of Co single atoms and few-atom clusters. Furthermore, the fact that PtCo-NC-3 shows a weaker white line and a more intense pre-edge feature at Co K-edge than PtCo-NC-1 suggests that clustering of Pt and Co atoms was more prominent in PtCo-NC-3 than in PtCo-NC-1.

The relevant Fourier transform extended X-ray absorption fine structure (EXAFS) spectra of Pt are shown in Figure 3(c). Fitting of the EXAFS data (Table S4) yields a Pt-N bond of ca. 2.0 Å for the Pt-NC-1, PtCo-NC-1, Pt-NC-3, and PtCo-NC-3 samples [47], in agreement with results from the EELS study (Figure 2(b)). This is also consistent with results obtained from DFT calculations, where the Pt-N bond length is found to be 1.95 Å-2.08 Å in different configurations (Figure S1). Note that no Pt-Pt bond can be identified in the Pt-NC-1 and PtCo-NC-1 samples, consistent with atomic dispersion of Pt within the carbon skeletons. Furthermore, the coordination number (CN) of Pt-N is estimated to be 3.5 for Pt-NC-1 and 3.6 for PtCo-NC-1, suggesting that Pt is mostly embedded with the carbon matrix in the form of PtN_3~4_ (Figure 1(d)).

For Pt-NC-3 and PtCo-NC-3, an additional bond can also be resolved at ca. 2.65 Å, which likely arises from the Pt-Pt/Pt-Co linkages—note that for the Pt foil, the Pt-Pt bond length is 2.76 Å, with a CN of 12 [48, 49]. For Pt-NC-3, the CN of Pt-N is estimated to be 2.9, and that for Pt-Pt is ca. 2.0, consistent with partial clustering in the sample forming nitrogen-terminated Pt few-atom clusters. For PtCo-NC-3, the CN is 1.7 for Pt-N, 5.0 for Pt-Pt, and 3.0 for Pt-Co, suggesting somewhat enhanced clustering in the sample as compared to Pt-NC-3. From the CN values, the average cluster size is calculated to be between 0.65 nm and 1.19 nm by the Calvin equation [50], in good agreement with the formation of Pt_2~7_ clusters, as observed in Figure 2(c).

The corresponding Co EXAFS spectra are included in Figure 3(d). For PtCo-NC-1, the Co-N bond is resolved at 2.03 Å (CN = 8) [51, 52] and Pt-Co bond at 2.81 Å (CN = 3) [53], suggesting atomic dispersion of Co atoms in the samples in the form of Pt-Co bonding linkages. For the PtCo-NC-3 sample, the Co-N bond can be identified at 1.94 Å (CN = 1.6), Co-Pt bond at 2.65 Å (CN = 1.8), and Co-Co at 2.50 Å (CN = 1.8). This is consistent with partial clustering of Pt and Co and the dispersion of Co into Pt few-atom clusters. In fact, one may notice that the CNs of Pt-N and Co-N in PtCo-3 are lower than that in PtCo-1. This is because in PtCo-3, some of the Pt and Co atoms form clusters and no longer bind to the nitrogen atoms.

**Scheme 2:**
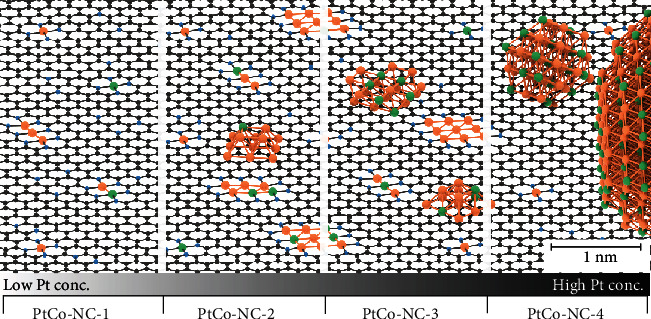
Proposed structures of the PtCo-NC samples based on the experimental characterization. Pt, Co, N, and C are denoted by orange, green, blue, and black, respectively.

Taken together, these results suggest a clear structural evolution from metal isolated atoms to few-atom clusters and to nanoparticles with increasing metal loadings in the sample synthesis (Scheme 2). For the samples prepared at low metal loadings (Pt-NC-1, PtCo-NC-1, Pt-NC-2, and PtCo-NC-2), isolated Pt atoms and Pt-Co pairs are dispersed within the carbon matrix. At higher metal loadings (Pt-NC-3 and PtCo-NC-3), partial clustering of Pt and Co occurred, with the sparse emergence of metal nanoparticles. At even higher metal loadings (Pt-NC-4 and PtCo-NC-4), nanoparticles become increasingly dominant. Further increase of the metal loadings led to the formation of abundant metal nanoparticles (Figure S8). The fitting of the EXAFS spectra can be found in Figure S9. Consistent results were obtained in X-ray diffraction (XRD) (Figure S10, Note S3), XPS (Figure S11–S14, Table S5–S9, Note S4), and Raman (Figure S15, Note S5) measurements.

### 2.3. Electrocatalytic Activity

**Figure 4:**
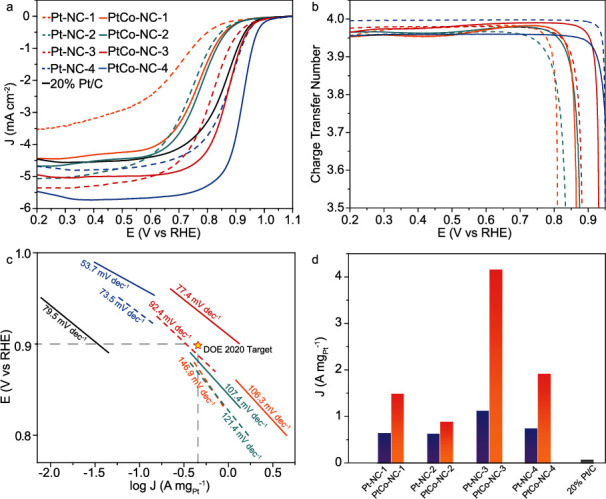
Electrocatalytic performance in oxygen reduction reaction. (a) LSV curves of (dashed curves) Pt-NC, (solid curves) PtCo-NC, and (black curve) commercial Pt/C in 0.1 M HClO_4_, and the corresponding (b) charge transfer number and (c) Tafel plots. In (c), the star marks the DOE 2020 target. (d) Mass activity of the samples and 20% Pt/C at +0.85 V vs. RHE. The blue and yellow columns represent the Pt-NC and PtCo-NC samples, respectively.

The electrocatalytic activity towards ORR is then assessed and compared. From Figure 4(a), one can see that all samples exhibit apparent electrocatalytic activity towards ORR in 0.1 M HClO_4_ at the catalyst loading of 0.16 mg cm^−2^. Yet, the activity of the Pt-NC series increases with samples prepared at increasing PtCl_4_ feeds, from Pt-NC-1 to Pt-NC-4. Specifically, Pt-NC-1, which consists of only isolated Pt single atoms and shows no voltammetric features for hydrogen adsorption/desorption (Figure S16), exhibits a relatively low activity, with a half-wave potential (*E*_1/2_) of +0.72 V vs. RHE, in comparison to +0.74 V for Pt-NC-2 and +0.82 V for Pt-NC-3, suggesting a large Pt domain is preferred for ORR. Pt-NC-4 is the best among the series, with *E*_1/2_ = +0.87 V and obvious hydrogen adsorption/desorption features (Figure S16). Interestingly, with the incorporation of Co into the samples, the ORR activity is enhanced drastically. For instance, PtCo-NC-1 shows an *E*_1/2_ of +0.77 V, ca. 50 mV more positive than that of Pt-NC-1. PtCo-NC-2 and PtCo-NC-3 also show a positive shift of 40 and 50 mV, as compared to Pt-NC-2 and Pt-NC-3, respectively. This enhancement can be ascribed to the Pt-Co coupling (no nanoparticle contribution in these samples). PtCo-NC-4 exhibit an even higher shift of 60 mV to +0.93 V, most likely because of the well-known Pt-Co alloying contribution [54–56]. As a reference, the sample containing only Co shows a very poor activity, indicating minimal contribution from Co SAs or Co NPs (Figure S17).

Moreover, the electron transfer number (*n*) involved in ORR of all samples is estimated to be over 3.95 (Figure 4(b)), suggesting that ORR adopted the 4-electron pathway on these catalysts, O_2_ + 4H^+^ + 4e^−^ → 2H_2_O, and the ORR activity is not limited by the second step (Equation (2)), which supports the conclusion that the first step (Equation (1)) is most likely the RDS (Figure 1). Remarkably, the PtCo-NC-3, Pt-NC-4, and PtCo-NC-4 samples all outperform commercial 20 wt% Pt/C, despite a significantly lower Pt loading (ca. 1 to 5 wt%). Figure 4(c) depicts the Tafel plots of the two series of samples. Indeed, the PtCo-NC samples show a smaller Tafel slope than the Pt-NC counterparts, indicating superior electron transfer kinetics in the former. Among them, PtCo-NC-3 shows a mass activity (at +0.9 V) that even exceeds the DOE 2020 target, with a low Tafel slope of 77.4 mV dec^−1^. The results are summarized in Table S10.

From Figure 4(d), one can see that the mass activity (at +0.85 V) of both the Pt-NC and PtCo-NC series exhibits a peak-shaped variation with the Pt content. Note that this Pt concentration range corresponds to a structural evolution from Pt isolated atoms to clusters and nanoparticles (Scheme 2). The results suggest that a minimum threshold of the Pt domain size needs to be reached for effective ORR electrocatalysis, and the incorporation of Co can significantly enhance the ORR performance by the unique Pt-Co coupling. Among the series, PtCo-NC-3 stands out as the best among the series, with a mass activity of 4.16 A mg_Pt_^−1^ at +0.85 V, 48 times better than that of commercial Pt/C.

It should be noted that in the above measurements, the overall catalyst loading was kept constant at 0.16 mg cm^−2^ among the Pt-NC and PtCo-NC series, corresponding to a clear variation of the Pt content in the catalysts, which has been known to impact the catalytic activity [57]. Thus, we carried out further measurements with the series of samples at a consistent Pt loading and compared the ORR performance. Figure S18 shows the corresponding electrochemical data with the series of Pt-NC and PtCo-NC samples at the same Pt loading of ca. 3 *μ*g cm^−2^. Specifically, in comparison to the studies in Figure 4(a), the catalyst loadings of Pt-NC-2 and PtCo-NC-2 were increased by 4 times, whereas the loadings of Pt-NC-4 and PtCo-NC-4 were reduced by 60% and that of commercial Pt/C by 90%. One can see that the *E*_1/2_ of Pt-NC-2 and PtCo-NC-2 exhibited a positive shift of 20-40 mV, while *E*_1/2_ of Pt-NC-4 and PtCo-NC-4 shifted negatively by 40 mV and that of commercial Pt/C by 170 mV. These observations suggest that the ORR activity decreased in the order of PtCo-NC-4 > Pt-NC-4 ~ PtCo-NC-2 > Pt-NC-2 > > Pt/C, consistent with the results obtained from Figure 4(a) where the catalyst loadings were the same, but the Pt contents were different, and further highlight the significant contribution of Pt-Co clustering to the enhancement of the ORR activity.

The stability of the samples was then tested. PtCo-NC-2 was found to exhibit a negative shift of only 30 mV after 5000 voltammetric cycles, a performance comparable to that of PtCo-NC-4 (Figure S19). This indicates that the stability of Pt single atoms/few-atom clusters was similar to that of small nanoparticles and drastically better than that of commercial Pt/C [4, 15]. Yet in the poisoning tests (Figure S20), the samples displayed markedly different behaviors. For PtCo-NC-4, which mainly contained PtCo alloy nanoparticles, the ORR activity was drastically reduced upon the addition of SCN^−^, whereas minimal impacts were observed after EDTA treatment. By contrast, for PtCo-NC-2 (consisting of few-atom clusters and single atoms), the ORR electrocatalysis was significantly impeded by both SCN^−^ and EDTA. Note that SCN^−^ nondiscriminatorily poisoned single atoms, few-atom clusters, and nanoparticles, whereas EDTA attacked predominantly single-metal atoms. Therefore, the observed discrepancy of the poisoning effects further confirms the significant contribution of PtCo single atoms/few-atom clusters to the ORR activity in ultralow Pt loading PtCo-NC.

## 3. Conclusion

In summary, two significant findings were obtained in this study. First, Pt_~9_ clusters were found to represent the threshold domain size of platinum towards ORR electrocatalysis in acid, whereas smaller clusters exhibited only limited ORR activity, as compared to conventional nanoparticles and bulk forms. Second, the electrocatalytic activity of few-atom Pt clusters can be markedly enhanced by clustering with Co atomic species, as clearly manifested in both theoretical calculations and experimental measurements, and Pt_2 ~ 7_ clusters doped with Co atoms were found to be the optimal sample, with a mass activity that was almost 50 times that of commercial Pt/C. This was accounted for by the formation of Pt-Co bonding interactions that facilitated the adsorption of important oxygen intermediates. Results from this study highlight the fundamental significance of atomic alloying in diminishing the threshold size of precious metal catalysts to the few-atom cluster level. Further enhancement of the electrocatalytic activity can be achieved with an accurate control of the Pt : Co ratio on the cluster level, which calls for the development of effective synthetic methods for structural engineering of the alloy clusters. The minimization of catalyst cost and remarkable enhancement of electrocatalytic performance is anticipated to play a critical role in the eventual practical implementation of electrochemical energy technologies.

## 4. Methods

### 4.1. Synthesis of Tellurium Nanowires

Te NWs were prepared by following a procedure described previously [58]. In a typical experiment, 0.1844 g of Na_2_TeO_3_ and 2 g of polyvinylpyrrolidone were dissolved in 65 mL of nanopure water under vigorous stirring, into which were then injected 3.3 mL of N_2_H_4_ and 6.7 mL of ammonia. The solution was transferred to a 100 mL Teflon lined autoclave container and heated at 180°C for 3 h. The autoclave was cooled down naturally and stored in a 4°C refrigerator for further use.

### 4.2. Synthesis of Te Nanowires Coated with Melamine-Formaldehyde Resin

The preparation of core-shell nanofibers (Te-MF) consisting of Te nanowires coated with an MF resin shell has been detailed previously [36, 42]. In brief, 10 mL of Te NWs was centrifuged at 3000 rpm for 2 min with the addition of acetone as a precipitation agent. After washing with water and ethanol for 3 times, Te NWs were dispersed in 10 mL of water. Separately, 0.126 g of melamine in 10 mL of water was added into a 50 mL round-bottom flask, and the solution was heated at 90°C under magnetic stirring, into which were then injected the Te NWs solution, 20 *μ*L of 0.2 M NaOH, and 0.53 mL of formaldehyde. The solution was heated at 90°C for 7 h before being cooled down naturally. The product was collected by centrifugation at 5000 rpm for 5 min, washed with water and ethanol, and dried in a freeze dryer for 24 h.

### 4.3. Synthesis of Pt- and PtCo-Doped Carbon Nanowires

In a typical experiment, 100 mg of the Te-MF nanofibers obtained above was dispersed into 30 mL of nanopure water under magnetic stirring at 350 rpm and heated at 50°C, into which was then added a varied amount of PtCl_4_ (0.5, 1, 2, and 6 mg). The reaction was run for 24 h, and the color of the solution was found to change from blue to pale white. The solids were then collected by centrifugation and rinsed with water for several times. The obtained product was equally divided into two parts. One part was directly dried with a freeze drier, while the other was mixed with a certain amount of CoCl_2_·6H_2_O (0.25, 0.5, 1, and 3 mg) and dried with a freeze drier. The mixtures were placed in a tube furnace and heated at 900°C for 3 h under a nitrogen flow of 200 cc min^−1^. The resulting samples were denoted as Pt-NC-*y* or PtCo-NC-*y* with *y* = 1, 2, 3, and 4.

### 4.4. Characterization

TEM images and EELS data were acquired with a Nion U-HERMS200 microscope operated at 60 kV. HAADF-STEM studies were carried out with a probe semiangle of 35 mrad at a spatial resolution of 0.11 nm. For EELS measurements, half convergence angle was set at 20 mrad, and a current was set at 150 pA with a dispersion of 0.268 eV channel^−1^. The integral time for spectral collection of the Pt signals was 2 s, and that of C, N signal was 12 s. High-resolution TEM (HRTEM) studies and elemental mapping based on energy-dispersive X-ray spectroscopy (EDS) were acquired with a FEI Tecnai G2 TF20 transmission electron microscope operated at 200 kV. ICP-OES measurements were carried out with a SPECTRO BLUE SOP instrument. XRD studies were performed with a SmartLab 9 KW XRD system. Raman spectra were collected with a Laser Microscopic Confocal Raman Spectrometer. XPS data were acquired with a PHI-5702 XPS instrument.

### 4.5. X-Ray Absorption Spectroscopy

Co K-edge and Pt L_3_-edge XAS data were collected at the CLS@APS (Sector 20-BM) beamline at the Advanced Photon Source (operating at 7.0 GeV) in Argonne National Laboratory, Chicago, IL, USA. Solid powder samples were loaded onto a Kapton tape and folded to ensure adequate signals. Samples were measured in the fluorescence mode simultaneously with each element foil reference. All measurements were conducted at room temperature and ambient pressure. EXAFS data was transformed and normalized into k- and R-space using the Athena program following conventional procedures. A k weighting of 2 was used to obtain all FT-EXAFS spectra. The k-range used for each sample is as follows: 3.9–8.7 Å^−1^ for Pt-NC-1, 2.5–10.0 Å^−1^ for Pt-NC-3, 3.0–10.7 Å^−1^ for PtCo-NC-1-Co, 2.6–11.0 Å^−1^ for PtCo-NC-1-Pt, 3.3–11.8 Å^−1^ for PtCo-NC-3-Co, and 3.4–10.3 Å^−1^ for PtCo-NC-3-Pt. The R-range used for each element is as follows: 1.0-3.0 Å for Pt-NC-1, 1.0–3.4 Å for Pt-NC-3, 1.0–3.0 Å for PtCo-NC-1-Co, 1.0-2.4 Å for PtCo-NC-1-Pt, 1.0–3.4 Å for PtCo-NC-3-Co, and 1.0–3.6 Å for PtCo-NC-3-Pt. Self-consistent multiple-scattering calculations were performed using the FEFF6 program to obtain the scattering amplitudes and phase-shift functions used to fit various scattering paths with the Artemis program. In the fitting of PtCo-NC-1 Co K-edge, the *E*_0_ values were correlated together to minimize the number of independent values, allowing reliable fitting results to be obtained. For the PtCo-NC-3 sample, the Co K-edge and Pt L_3_-edge were fitted simultaneously. The Pt-Co and Co-Pt bond distances were correlated, as well as the *E*_0_ and *σ*^2^ values for each individual element.

### 4.6. Electrochemistry

Electrochemical measurements were carried out with a CHI 710 electrochemical workstation in a conventional three-electrode configuration. A Ag/AgCl electrode in 0.1 M KCl was used as the reference electrode and a graphite rod as the counter electrode. The Ag/AgCl reference was calibrated against a reversible hydrogen electrode (RHE), and all potentials in the present study were referenced to this RHE. To prepare catalyst inks, 4 mg of the catalysts obtained above was added into 1 mL of ethanol and 10 *μ*L of Nafion solution under sonication to form a homogeneous dispersion. 10 *μ*L of the ink was then dropcast onto a clean glassy carbon disk electrode (surface area 0.246 cm^2^) at the catalyst loading of 0.16 mg cm^−2^. iR compensation was set at 90% of solution resistance in all measurements. The number of electron transfer is calculated by *n* = 4*i*_d_/*i*_d_ + (*i*_r_/*N*), where *i*_d_ and *i*_r_ are the disk and ring currents, respectively, and *N* is the collection efficiency of the ring electrode (0.37). The ORR performance was also tested with the series of samples at the same Pt loading of ca. 3 *μ*g cm^−2^. The NaSCN poisoning test was carried out in a solution of 0.1 M HClO_4_ and 10 mM NaSCN. The EDTA poisoning test was carried out after treating the catalyst samples with 10 mM EDTA and 1 M KOH at 60°C overnight.

### 4.7. Computational Method

Computation studies were carried out with open-source plane wave code Quantum ESPRESSO [59]. A two-dimensional 8 × 8 supercell was built with a vacuum thickness set at 14 Å to avoid interaction between periodic images. The ultrasoft pseudopotential [60] was adopted with kinetic and charge density cutoff energy at 40 Ry and 240 Ry, respectively. A 2 × 2 × 1 Monkhorst-Pack K-point grid was sampled to converge the total energy to 10^−3^ eV. Marzari-Vanderbilt smearing [61] was adopted with 0.01 Ry. For geometric relaxation, the electronic energy was converged to 10^−6^ Ry and force converged to 10^−4^ a.u., respectively. Density functional perturbation theory (DFPT) [62] was employed to compute the vibration frequencies of surface species for zero-point energy (ZPE) and entropy contribution, similar as previous work [36, 42]. Atomic charge was analyzed based on Bader charge partitioning scheme [63].

## Supplementary Materials

Supplementary Materials Figure S1: structural configurations of Pt and PtCo in varied domain size embedded in nitrogen-doped graphene. Figure S2: linear trimers of metals in nitrogen-doped graphene. Figure S3: structures of Pt_5_Co_1_∗, Pt_1_Co_8_, and Pt_8_Co_1_∗ with the center Pt atoms highlighted by the blue arrow. Figure S4: OOH∗ intermediate adopts a bridge binding fashion on a PtCo dimer. Figure S5: PtCo with or without a direct bonding linkage in a nitrogen-doped graphene matrix. Figure S6: TEM images of the Pt-NC series. Figure S7: TEM images of the PtCo-NC series. Figure S8: TEM images of (a) Pt and (b) PtCo nanoparticles on carbon nanowires. Figure S9: EXAFS spectra of the Pt-NC and PtCo-NC samples. Figure S10: XRD patterns of the Pt-NC and PtCo-NC samples. Figure S11: XPS full survey spectra of the Pt-NC and PtCo-NC samples. Figure S12: high-resolution XPS scans of the C 1 s electrons of the Pt-NC and PtCo-NC samples. Figure S13: high-resolution XPS scans of the N 1 s electrons of the Pt-NC and PtCo-NC samples. Figure S14: (a) XPS spectra of the Pt 4f electrons in Pt-NC-1, Pt-NC-2, Pt-NC-3, and Pt-NC-4. (b) XPS spectra of the Pt 4f electrons in PtCo-NC-1, PtCo-NC-2, PtCo-NC-3, and PtCo-NC-4. (c) XPS spectra of the Co 2p electrons in PtCo-NC-1, PtCo-NC-2, PtCo-NC-3, and PtCo-NC-4. Figure S15: Raman spectra of the Pt-NC and PtCo-NC samples. Figure S16: CV curves of the Pt-NC and PtCo-NC samples in 0.1 M HClO_4_ at the scan rate of 50 mV s^−1^. Figure S17: ORR LSV curve and charge transfer number of Co,N-codoped C nanowires. Figure S18: ORR polarization curves of the series of samples at the same Pt loading of ca. 3 *μ*gPt cm^−2^. Figure S19: stability tests of select samples. Figure S20: poisoning test of (a) PtCo-NC-2 and (b) PtCo-NC-4. Table S1: Gibbs free energies of each step in ORR for a range of Pt-NC and PtCo-NC configurations as shown in Figure S1 at the potential of +0.9 V. Table S2: Bader charges of selected structures in Figure S1. Table S3: elemental analysis of the Pt-NC and PtCo-NC samples by ICP-OES measurements. Table S4: EXAFS fitting results. Table S5: fitting results of the Pt 4f XPS data. Table S6: fitting results of the Co 2p XPS data. Table S7: Pt to Co atomic ratio by XPS measurements. Table S8: fitting results of the C 1 s XPS data. Table S9: fitting results of the N 1 s XPS data. Table S10: summary of the ORR activity.

## References

[1] Pegis M. L., Wise C. F., Martin D. J., Mayer J. M. (2018). Oxygen reduction by homogeneous molecular catalysts and electrocatalysts. *Chemical Reviews*.

[2] Kulkarni A., Siahrostami S., Patel A., Nørskov J. K. (2018). Understanding catalytic activity trends in the oxygen reduction reaction. *Chemical Reviews*.

[3] Cheng F., Chen J. (2012). Metal-air batteries: from oxygen reduction electrochemistry to cathode catalysts. *Chemical Society Reviews*.

[4] Zhou Z.-Y., Kang X., Song Y., Chen S. (2012). Enhancement of the electrocatalytic activity of Pt nanoparticles in oxygen reduction by chlorophenyl functionalization. *Chemical Communications*.

[5] Nie Y., Li L., Wei Z. (2015). Recent advancements in Pt and Pt-free catalysts for oxygen reduction reaction. *Chemical Society Reviews*.

[6] Xiong Y., Yang Y., DiSalvo F. J., Abruña H. D. (2019). Metal-organic-framework-derived Co-Fe bimetallic oxygen reduction electrocatalysts for alkaline fuel cells. *Journal of the American Chemical Society*.

[7] Shen H., Gracia-Espino E., Ma J. (2017). Synergistic effects between atomically dispersed Fe-N-C and C-S-C for the oxygen reduction reaction in acidic media. *Angewandte Chemie-International Edition*.

[8] Chen P., Zhou T., Xing L. (2017). Atomically dispersed iron–nitrogen species as electrocatalysts for bifunctional oxygen evolution and reduction reactions. *Angewandte Chemie International Edition*.

[9] Frank B., Kahl P., Podbiel D. (2017). Short-range surface plasmonics: localized electron emission dynamics from a 60-nm spot on an atomically flat single-crystalline gold surface. *Science Advances*.

[10] Wang Z., Yao X., Kang Y., Miao L., Xia D., Gan L. (2019). Structurally ordered low-Pt intermetallic electrocatalysts toward durably high oxygen reduction reaction activity. *Advanced Functional Materials*.

[11] Wang R., Wang L., Zhou W. (2018). Ni2P entwined by graphite layers as a low-Pt electrocatalyst in acidic media for oxygen reduction. *ACS Applied Materials & Interfaces*.

[12] Saha S., Cabrera Rodas J. A., Tan S., Li D. (2018). Performance evaluation of platinum-molybdenum carbide nanocatalysts with ultralow platinum loading on anode and cathode catalyst layers of proton exchange membrane fuel cells. *Journal of Power Sources*.

[13] Liu Y., Kelly T. G., Chen J. G., Mustain W. E. (2013). Metal carbides as alternative electrocatalyst supports. *ACS Catalysis*.

[14] Li Z., Niu W., Yang Z. (2020). Boosting alkaline hydrogen evolution: the dominating role of interior modification in surface electrocatalysis. *Energy & Environmental Science*.

[15] Yamamoto K., Imaoka T., Chun W.-J. (2009). Size-specific catalytic activity of platinum clusters enhances oxygen reduction reactions. *Nature Chemistry*.

[16] Peng Y., Lu B., Chen S. (2018). Carbon-supported single atom catalysts for electrochemical energy conversion and storage. *Advanced Materials*.

[17] Song X., Li N., Zhang H., Wang H., Wang L., Bian Z. (2019). Promotion of hydrogen peroxide production on graphene-supported atomically dispersed platinum: effects of size on oxygen reduction reaction pathway. *Journal of Power Sources*.

[18] Choi C. H., Kim M., Kwon H. C. (2016). Tuning selectivity of electrochemical reactions by atomically dispersed platinum catalyst. *Nature Communications*.

[19] Yang S., Tak Y. J., Kim J., Soon A., Lee H. (2017). Support effects in single-atom platinum catalysts for electrochemical oxygen reduction. *ACS Catalysis*.

[20] Yang S., Kim J., Tak Y. J., Soon A., Lee H. (2016). Single-atom catalyst of platinum supported on titanium nitride for selective electrochemical reactions. *Angewandte Chemie International Edition*.

[21] Li T., Liu J., Song Y., Wang F. (2018). Photochemical solid-phase synthesis of platinum single atoms on nitrogen-doped carbon with high loading as bifunctional catalysts for hydrogen evolution and oxygen reduction reactions. *ACS Catalysis*.

[22] Dong J.-C., Zhang X.-G., Briega-Martos V. (2019). In situ Raman spectroscopic evidence for oxygen reduction reaction intermediates at platinum single-crystal surfaces. *Nature Energy*.

[23] Mayrhofer K. J. J., Blizanac B. B., Arenz M., Stamenkovic V. R., Ross P. N., Markovic N. M. (2005). The impact of geometric and surface electronic properties of Pt-catalysts on the particle size effect in electrocatalysis. *Journal of Physical Chemistry B*.

[24] Stamenkovic V. R., Fowler B., Mun B. S. (2007). Improved oxygen reduction activity on Pt3Ni(111) via increased surface site availability. *Science*.

[25] Liu C., Ma Z., Cui M. (2018). Favorable core/shell interface within Co2P/Pt nanorods for oxygen reduction electrocatalysis. *Nano Letters*.

[26] Zhang S., Zhang X., Jiang G. (2014). Tuning nanoparticle structure and surface strain for catalysis optimization. *Journal of the American Chemical Society*.

[27] Wang G., Yang Z., du Y., Yang Y. (2019). Programmable exposure of Pt active facets for efficient oxygen reduction. *Angewandte Chemie International Edition*.

[28] Stamenkovic V., Mun B. S., Mayrhofer K. J. J. (2006). Changing the activity of electrocatalysts for oxygen reduction by tuning the surface electronic structure. *Angewandte Chemie International Edition*.

[29] Strasser P., Koh S., Anniyev T. (2010). Lattice-strain control of the activity in dealloyed core–shell fuel cell catalysts. *Nature Chemistry*.

[30] Liu H.-l., Nosheen F., Wang X. (2015). Noble metal alloy complex nanostructures: controllable synthesis and their electrochemical property. *Chemical Society Reviews*.

[31] Oezaslan M., Strasser P. (2011). Activity of dealloyed PtCo3 and PtCu3 nanoparticle electrocatalyst for oxygen reduction reaction in polymer electrolyte membrane fuel cell. *Journal of Power Sources*.

[32] Chong L., Wen J., Kubal J. (2018). Ultralow-loading platinum-cobalt fuel cell catalysts derived from imidazolate frameworks. *Science*.

[33] Chai G. L., Hou Z., Shu D. J., Ikeda T., Terakura K. (2014). Active sites and mechanisms for oxygen reduction reaction on nitrogen-doped carbon alloy catalysts: Stone-Wales defect and curvature effect. *Journal of the American Chemical Society*.

[34] Viswanathan V., Hansen H. A., Rossmeisl J., Nørskov J. K. (2012). Universality in oxygen reduction electrocatalysis on metal surfaces. *ACS Catalysis*.

[35] Peng Y., Lu B., Wang N., Li L., Chen S. (2017). Impacts of interfacial charge transfer on nanoparticle electrocatalytic activity towards oxygen reduction. *Physical Chemistry Chemical Physics*.

[36] Lu B., Smart T. J., Qin D. (2017). Nitrogen and iron-codoped carbon hollow nanotubules as high-performance catalysts toward oxygen reduction reaction: a combined experimental and theoretical study. *Chemistry of Materials*.

[37] Li M., Zhang L., Xu Q., Niu J., Xia Z. (2014). N-doped graphene as catalysts for oxygen reduction and oxygen evolution reactions: theoretical considerations. *Journal of Catalysis*.

[38] Darby M. T., Stamatakis M., Michaelides A., Sykes E. C. H. (2018). Lonely atoms with special gifts: breaking linear scaling relationships in heterogeneous catalysis with single-atom alloys. *Journal of Physical Chemistry Letters*.

[39] He Z., Yang Y., Liu J.-W., Yu S. H. (2017). Emerging tellurium nanostructures: controllable synthesis and their applications. *Chemical Society Reviews*.

[40] Yang Y., Liu J.-W., Yu S.-H. (2016). Coiling ultrathin tellurium nanowires into nanorings by Pickering emulsion. *Chemical Communications*.

[41] Zhang W., Wu Z. Y., Jiang H. L., Yu S. H. (2014). Nanowire-directed templating synthesis of metal-organic framework nanofibers and their derived porous doped carbon nanofibers for enhanced electrocatalysis. *Journal of the American Chemical Society*.

[42] Lu B., Guo L., Wu F. (2019). Ruthenium atomically dispersed in carbon outperforms platinum toward hydrogen evolution in alkaline media. *Nature Communications*.

[43] Jamil R., Sohail M., Baig N., Ansari M. S., Ahmed R. (2019). Synthesis of hollow Pt-Ni nanoboxes for highly efficient methanol oxidation. *Scientific Reports*.

[44] Qiao B., Wang A., Yang X. (2011). Single-atom catalysis of CO oxidation using Pt-1/FeOx. *Nature Chemistry*.

[45] Wei H., Liu X., Wang A. (2014). FeOx-supported platinum single-atom and pseudo-single-atom catalysts for chemoselective hydrogenation of functionalized nitroarenes. *Nature Communications*.

[46] Itoi H., Nishihara H., Kobayashi S. (2017). Fine dispersion of Pt4–5 subnanoclusters and Pt single atoms over porous carbon supports and their structural analyses with X-ray absorption spectroscopy. *Journal of Physical Chemistry C*.

[47] Sun M., Ji J., Hu M. (2019). Overwhelming the performance of single atoms with atomic clusters for platinum-catalyzed hydrogen evolution. *ACS Catalysis*.

[48] Liu C. G., Zhang L. L., Chen X. M. (2019). CO oxidation over the polyoxometalate-supported single-atom catalysts M-1/POM (Fe, Co, Mn, Ru, Rh, Os, Ir, and Pt; POM = [PW12O40](3-)): a computational study on the activation of surface oxygen species. *Dalton Transactions*.

[49] Kaito T., Tanaka H., Mitsumoto H. (2016). In situ X-ray absorption fine structure analysis of PtCo, PtCu, and PtNi alloy electrocatalysts: the correlation of enhanced oxygen reduction reaction activity and structure. *The Journal of Physical Chemistry C*.

[50] Frenkel A. I., Yevick A., Cooper C., Vasic R. (2011). Modeling the structure and composition of nanoparticles by extended X-ray absorption fine-structure spectroscopy. *Annual Review of Analytical Chemistry*.

[51] Wang J., Huang Z., Liu W. (2017). Design of N-coordinated dual-metal sites: a stable and active Pt-free catalyst for acidic oxygen reduction reaction. *Journal of the American Chemical Society*.

[52] Han Y., Wang Y.-G., Chen W. (2017). Hollow N-doped carbon spheres with isolated cobalt single atomic sites: superior electrocatalysts for oxygen reduction. *Journal of the American Chemical Society*.

[53] Kanan M. W., Yano J., Surendranath Y., Dincă M., Yachandra V. K., Nocera D. G. (2010). Structure and valency of a cobalt−phosphate water oxidation catalyst determined by in situ X-ray spectroscopy. *Journal of the American Chemical Society*.

[54] Wang D., Xin H. L., Hovden R. (2013). Structurally ordered intermetallic platinum–cobalt core–shell nanoparticles with enhanced activity and stability as oxygen reduction electrocatalysts. *Nature Materials*.

[55] Guo S., Li D., Zhu H. (2013). FePt and CoPt nanowires as efficient catalysts for the oxygen reduction reaction. *Angewandte Chemie-International Edition*.

[56] Wang C., van der Vliet D., Chang K.-C. (2009). Monodisperse Pt3Co nanoparticles as a catalyst for the oxygen reduction reaction: size-dependent activity. *The Journal of Physical Chemistry C*.

[57] Mayrhofer K. J. J., Strmcnik D., Blizanac B. B., Stamenkovic V., Arenz M., Markovic N. M. (2008). Measurement of oxygen reduction activities via the rotating disc electrode method: from Pt model surfaces to carbon-supported high surface area catalysts. *Electrochimica Acta*.

[58] Liu J.-W., Zhu J.-H., Zhang C.-L., Liang H. W., Yu S. H. (2010). Mesostructured assemblies of ultrathin superlong tellurium nanowires and their photoconductivity. *Journal of the American Chemical Society*.

[59] Giannozzi P., Baroni S., Bonini N. (2009). Quantum ESPRESSO: a modular and open-source software project for quantum simulations of materials. *Journal of Physics-Condensed Matter*.

[60] Garrity K. F., Bennett J. W., Rabe K. M., Vanderbilt D. (2014). Pseudopotentials for high-throughput DFT calculations. *Computational Materials Science*.

[61] Marzari N., Vanderbilt D., de Vita A., Payne M. C. (1999). Thermal contraction and disordering of the Al(110) surface. *Physical Review Letters*.

[62] Baroni S., de Gironcoli S., Dal Corso A., Giannozzi P. (2001). Phonons and related crystal properties from density-functional perturbation theory. *Reviews of Modern Physics*.

[63] Yu M., Trinkle D. R. (2011). Accurate and efficient algorithm for Bader charge integration. *The Journal of Chemical Physics*.

[64] Towns J., Cockerill T., Dahan M. (2014). XSEDE: accelerating scientific discovery. *Computing in Science & Engineering*.

