# Supplementary material for: Oxygen Reduction Reaction Catalyzed by Carbon-Supported Platinum Few-Atom Clusters: Significant Enhancement by Doping of Atomic Cobalt
Source: Research (Wash D C). 2020 Nov 6;2020:9167829. doi: 10.34133/2020/9167829 (PMC7877387; doi:10.34133/2020/9167829)
Supplement: Supplementary Materials — Figure S1: structural configurations of Pt and PtCo in varied domain size embedded in nitrogen-doped graphene. Figure S2: linear trimers of metals in nitrogen-doped graphene. Figure S3: structures of Pt5Co1∗, Pt1Co8, and Pt8Co1∗ with the center Pt atoms highlighted by the blue arrow. Figure S4: OOH∗ intermediate adopts a bridge binding fashion on a PtCo dimer. Figure S5: PtCo with or without a direct bonding linkage in a nitrogen-doped graphene matrix. Figure S6: TEM images of the Pt-NC series. Figure S7: TEM images of the PtCo-NC series. Figure S8: TEM images of (a) Pt and (b) PtCo nanoparticles on carbon nanowires. Figure S9: EXAFS spectra of the Pt-NC and PtCo-NC samples. Figure S10: XRD patterns of the Pt-NC and PtCo-NC samples. Figure S11: XPS full survey spectra of the Pt-NC and PtCo-NC samples. Figure S12: high-resolution XPS scans of the C 1 s electrons of the Pt-NC and PtCo-NC samples. Figure S13: high-resolution XPS scans of the N 1 s electrons of the Pt-NC and PtCo-NC samples. Figure S14: (a) XPS spectra of the Pt 4f electrons in Pt-NC-1, Pt-NC-2, Pt-NC-3, and Pt-NC-4. (b) XPS spectra of the Pt 4f electrons in PtCo-NC-1, PtCo-NC-2, PtCo-NC-3, and PtCo-NC-4. (c) XPS spectra of the Co 2p electrons in PtCo-NC-1, PtCo-NC-2, PtCo-NC-3, and PtCo-NC-4. Figure S15: Raman spectra of the Pt-NC and PtCo-NC samples. Figure S16: CV curves of the Pt-NC and PtCo-NC samples in 0.1 M HClO4 at the scan rate of 50 mV s−1. Figure S17: ORR LSV curve and charge transfer number of Co,N-codoped C nanowires. Figure S18: ORR polarization curves of the series of samples at the same Pt loading of ca. 3 μgPt cm−2. Figure S19: stability tests of select samples. Figure S20: poisoning test of (a) PtCo-NC-2 and (b) PtCo-NC-4. Table S1: Gibbs free energies of each step in ORR for a range of Pt-NC and PtCo-NC configurations as shown in Figure S1 at the potential of +0.9 V. Table S2: Bader charges of selected structures in Figure S1. Table S3: elemental analysis of the Pt-NC and PtCo-NC sam [file 9167829.f1.pdf]

Supporting information

# Oxygen Reduction Reaction Catalyzed by Carbon-Supported Platinum Few-Atom Clusters: Significant Enhancement by Doping of Atomic Cobalt

**Bingzhang Lu,[1] Qiming Liu,[1] Forrest Nichols,[1] Rene Mercado,[1] David Morris,[2] Ning Li, [3,4] Peng Zhang,[2] Peng Gao,[3,4,5] Yuan Ping,[1] and Shaowei Chen[1,*]**

[1] *Department of Chemistry and Biochemistry, University of California, 1156 High Street, Santa Cruz, California 950564, USA.*

[2] *Department of Chemistry, Dalhousie University, 6274 Coburg Road, Halifax, Nova Scotia B3H 4R2, Canada*

[3] *International Center for Quantum Materials, School of Physics, Peking University, Beijing 100871, CHINA*

[4] *Electron Microscopy Laboratory, School of Physics, Peking University, Beijing 100871, CHINA*
[5] *Collaborative Innovation Centre of Quantum Matter, Beijing 100871, CHINA*

Correspondence should be addressed to Shaowei Chen; shaowei@ucsc.edu

**Table of Contents**


Figure S1 ....................(4)

Figure S2 ....................(5)

Figure S3 ....................(5)

Figure S4 ....................(5)

Figure S5 ....................(6)

Figure S6 ....................(6)

Figure S7 ....................(6)

Figure S8 ....................(7)

Figure S9 ....................(7)

Figure S10 ....................(8)

Figure S11 ....................(8)

Figure S12 ....................(9)

Figure S13 ....................(10)

Figure S14 ....................(10)

Figure S15 ....................(11)

Figure S16 ....................(11)

Figure S17 ....................(12)

Figure S18 ....................(12)

Figure S19 ....................(13)

Figure S20 ....................(13)

Table S1 ....................(14)

Table S2 ....................(15)

Table S3 ....................(15)

Table S4 ....................(15)

Table S5 ....................(16)

Table S6 ....................(16)

Table S7 ....................(16)

Table S8 ....................(16)

Table S9 ....................(17)

Table S10 ....................(17)

Note S1 ....................(18)

Note S2 ....................(18)

Note S3 ....................(18)

Note S4 ....................(19)

Note S5 ....................(19)

References ....................(20)

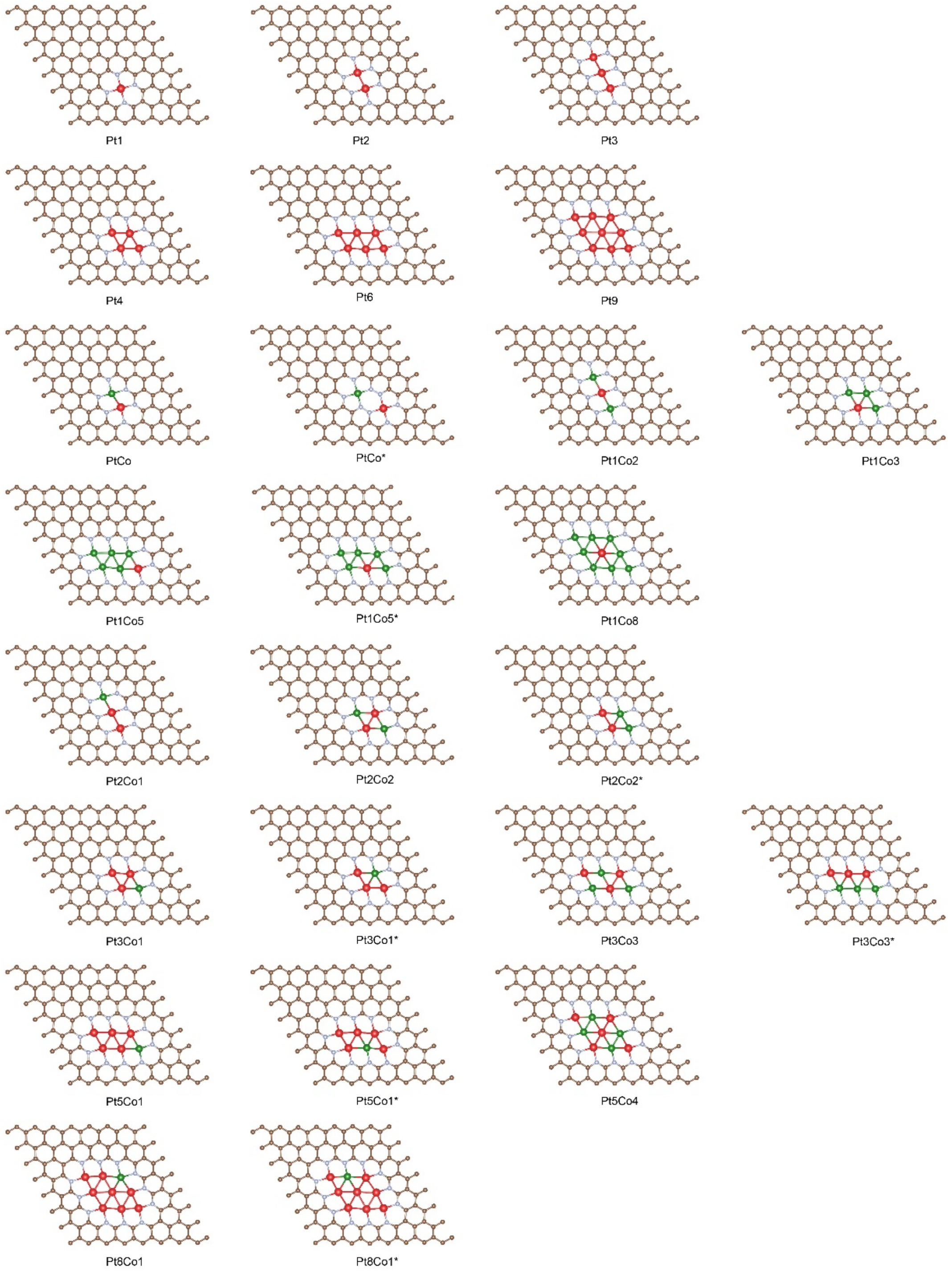


**Figure S1**. Structural configurations of Pt and PtCo in varied domain size embedded in nitrogen-doped graphene. The red, green, grey and brown balls represent Pt, Co, N and C atoms, respectively.

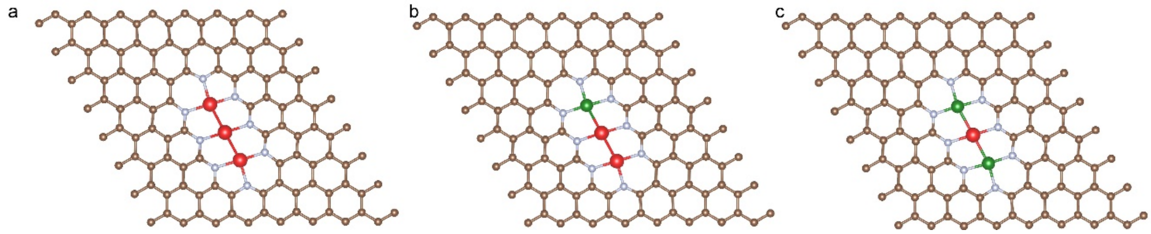


**Figure S2**. Linear trimers of metals in nitrogen-doped graphene: (a) $Pt_3$, (b) $Pt_2Co_1$, and (c) $Pt_1Co_2$. The red, green, grey and brown balls represent Pt, Co, N and C atoms, respectively.

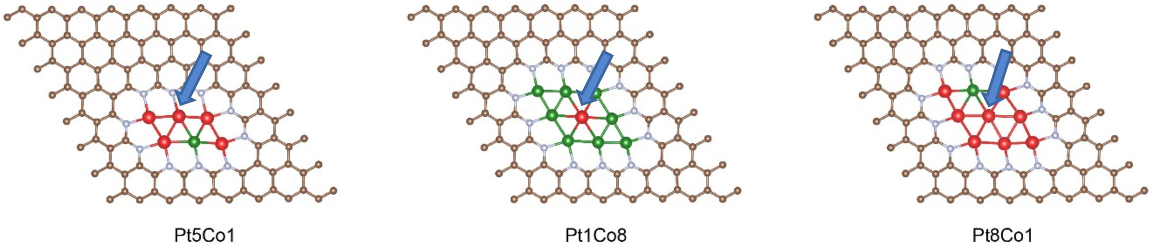


**Figure S3**. Structures of $Pt_5Co_1$*, $Pt_1Co_8$ and $Pt_8Co_1$* with the center Pt atoms highlighted by the blue arrow. The red, green, grey and brown balls represent Pt, Co, N and C atoms, respectively.

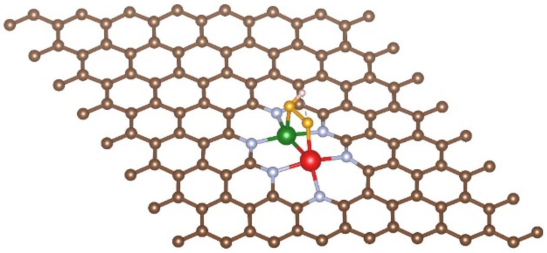

**Figure S4.** OOH* intermediate adopts a bridge binding fashion on a PtCo dimer.

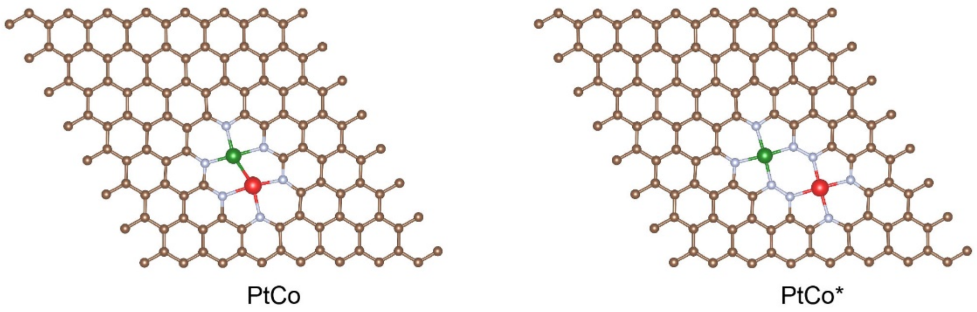


**Figure S5**. PtCo with or without a direct bonding linkage in a nitrogen-doped graphene matrix. The distance between Co (green) and Pt (red) is 2.27 Å for direct bonding (left) and 4.01 Å for non-direct bonding (right).

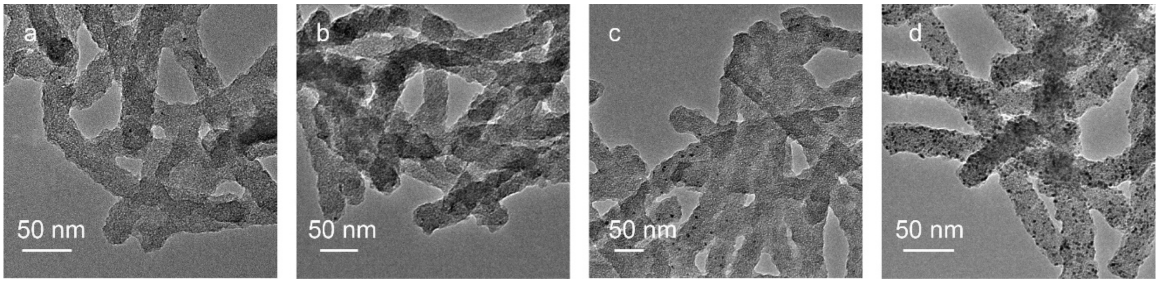


**Figure S6**. TEM images of the Pt-NC series: (a) Pt-NC-1, (b) Pt-NC-2, (c) Pt-NC-3 and (d) Pt-NC-4. Scale bars all 50 nm.

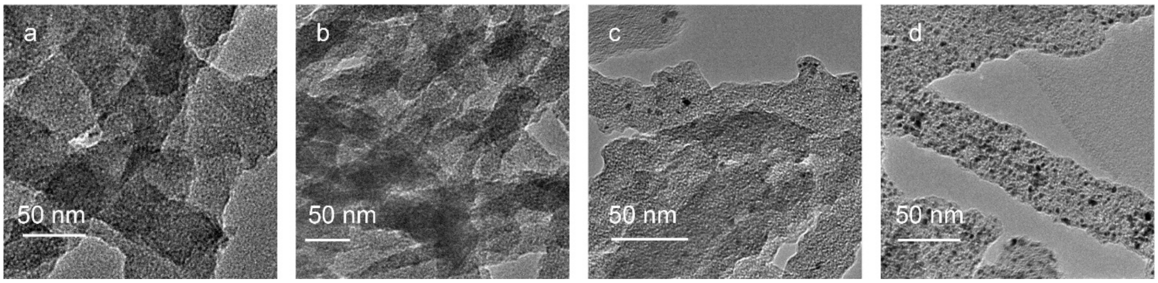


**Figure S7**. TEM images of the PtCo-NC series**:** (a) PtCo-NC-1, (b) PtCo-NC-2, (c) PtCo-NC-3 and (d) PtCo-NC-4. Scale bars all 50 nm.

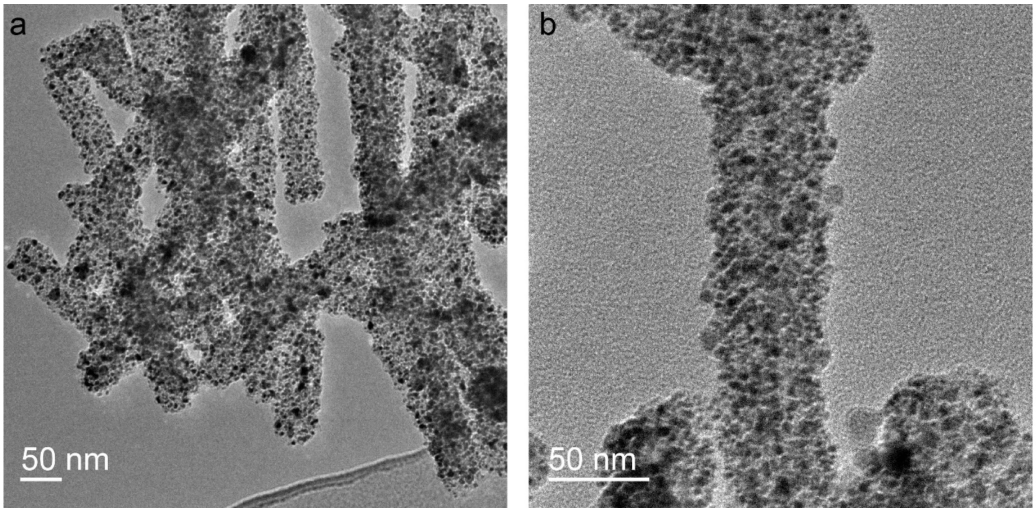


**Figure S8**. TEM images of (a) Pt and (b) PtCo nanoparticles on carbon nanowires. The samples are prepared with a Pt concentration 5 times than that of Pt-NC-4 (and PtCo-NC-4). Scale bars 50 nm.

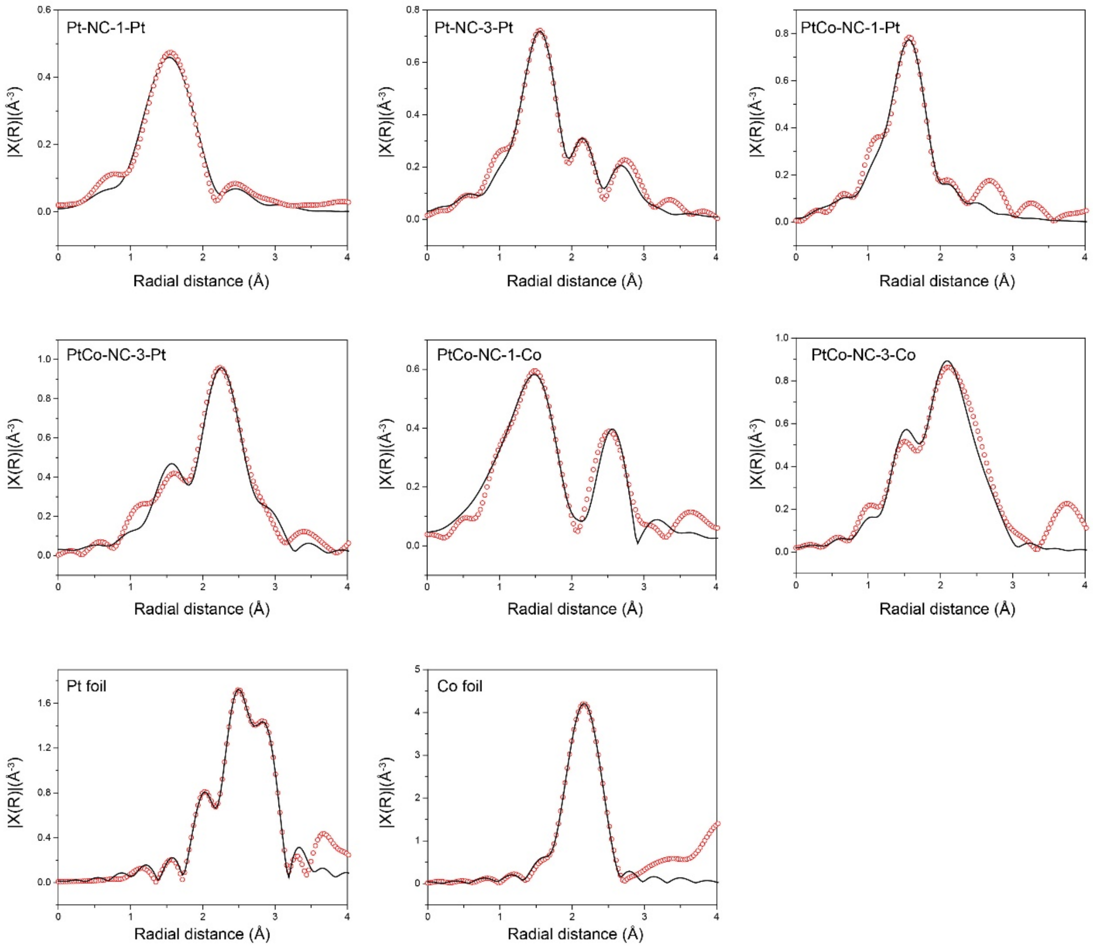


**Figure S9**. EXAFS spectra of the Pt-NC and PtCo-NC samples. Red circles are experimental data and black curves are best fits. The data for Pt and Co foils are also included as a reference.

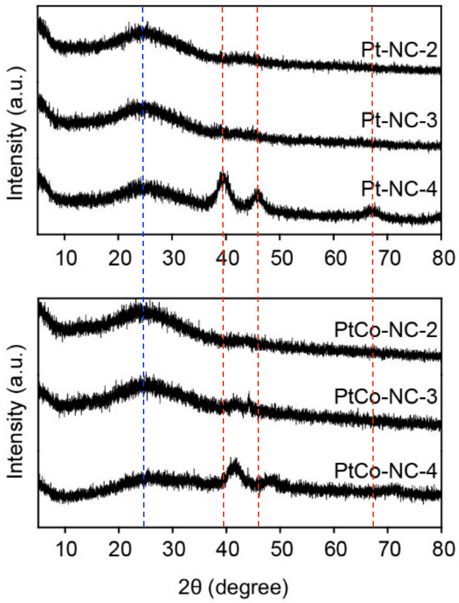


**Figure S10**. XRD patterns of the Pt-NC and PtCo-NC samples. The diffraction peak centered at ca. 25° (blue dashed line) is due to the (002) diffraction of graphitic carbon, whereas the peaks 40°, 46° and 67° (red dashed lines) observed with Pt-NC-4 are assigned to the (111), (200) and (220) diffractions of *fcc* Pt (card 00-001-1190). The latter peaks appear at slightly higher 2θ angles in the PtCo-NC-4 sample, indicating lattice shrinkage due to alloying between Pt and Co in the NPs.

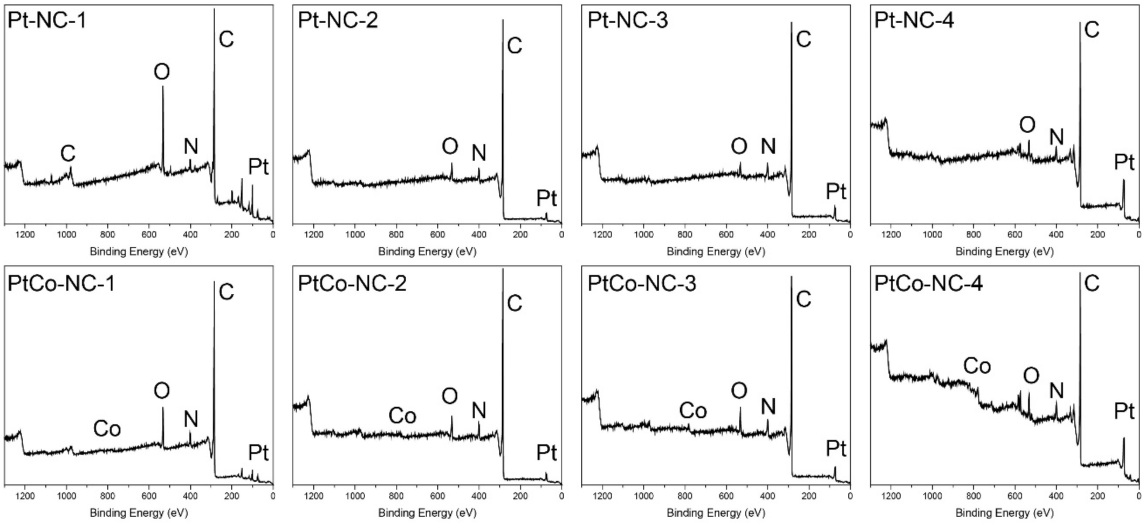


**Figure S11**. XPS full survey spectra of the Pt-NC and PtCo-NC samples. The elements of C, N, and Pt can be clearly identified with the Pt-NC and PtCo-NC series (with Co for the latter).

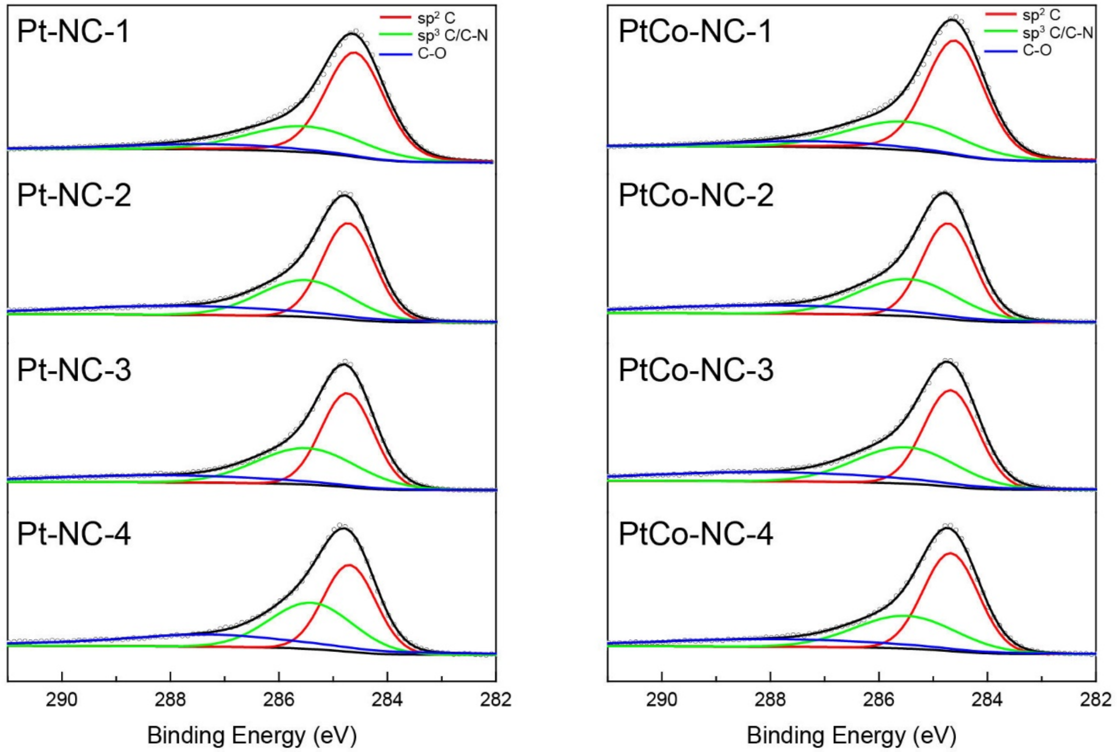


**Figure S12**. High-resolution XPS scans of the C 1s electrons of the Pt-NC and PtCo-NC samples. Grey circles are experimental data and colored curves are deconvolution fits.

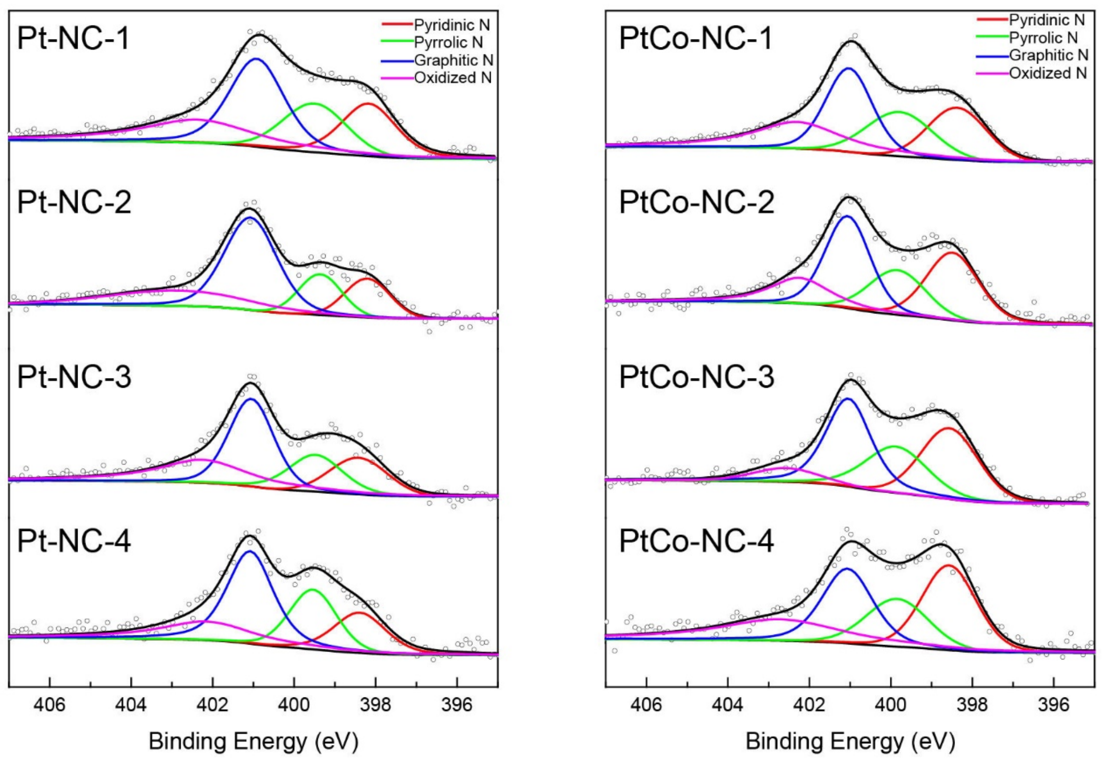


**Figure S13**. High-resolution XPS scans of the N 1s electrons of the Pt-NC and PtCo-NC samples. Grey circles are experimental data and colored curves are deconvolution fits.

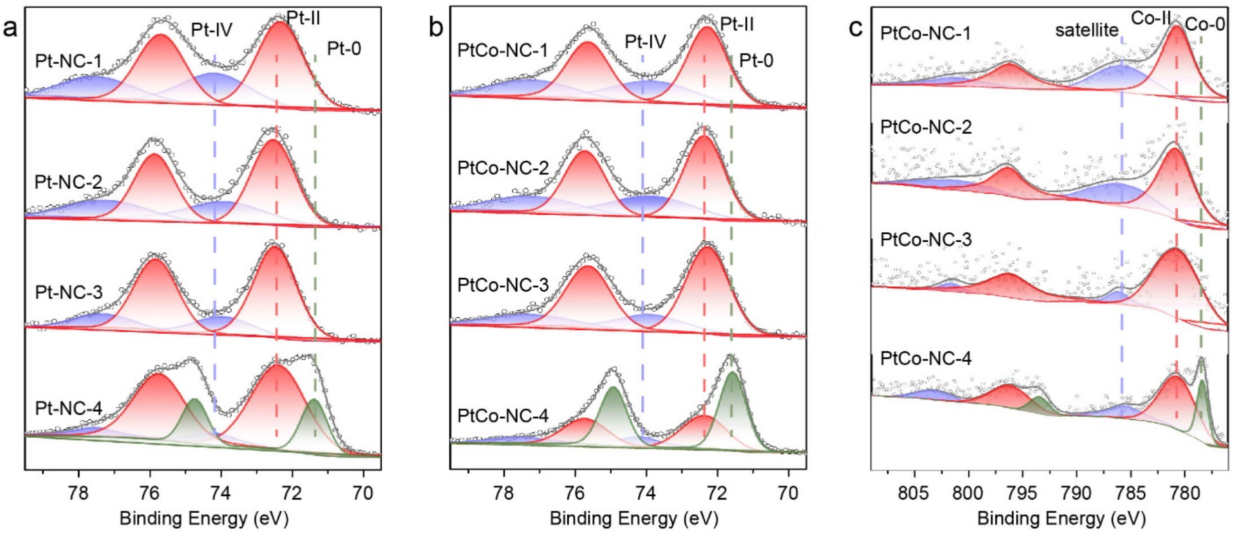


**Figure S14.** (a) XPS spectra of the Pt 4f electrons in Pt-NC-1, Pt-NC-2, Pt-NC-3 and Pt-NC-4. (b) XPS spectra of the Pt 4f electrons in PtCo-NC-1, PtCo-NC-2, PtCo-NC-3 and PtCo-NC-4. (c) XPS spectra of the Co 2p electrons in PtCo-NC-1, PtCo-NC-2, PtCo-NC-3 and PtCo-NC-4.

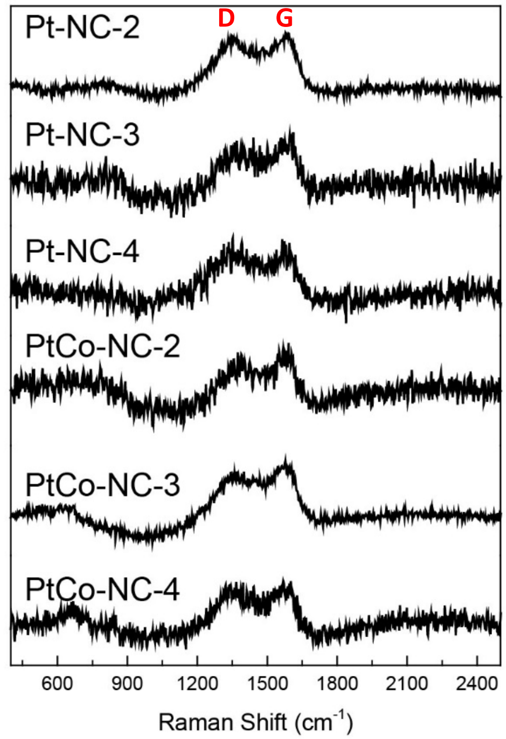


**Figure S15**. Raman spectra of the Pt-NC and PtCo-NC samples. The D and G bands can be readily identified with a similar band intensity ratio in all samples, suggesting a rather consistent carbon matrix.

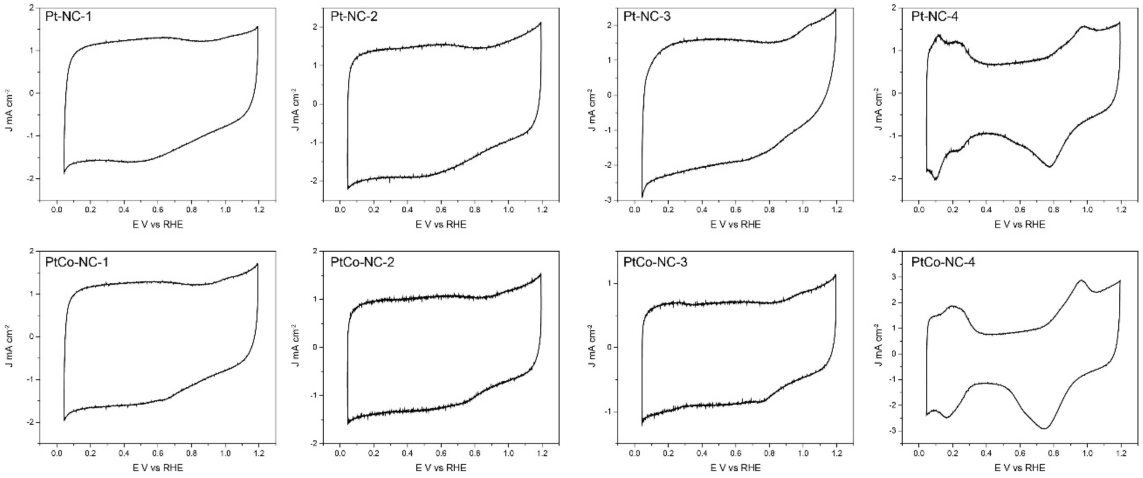


**Figure S16**. CV curves of the Pt-NC and PtCo-NC samples in 0.1 M $HClO_4$ at the scan rate of 50 mV $s^{-1}$. Note that only Pt-NC-4 and PtCo-NC-4 show apparent hydrogen adsorption/desorption voltammetric features, whereas the other samples display only featureless responses, consistent with the formation of only Pt isolated atoms/few-atom clusters in the latter, as compared to Pt NPs in Pt-NC-4 and PtCo-NC-4.

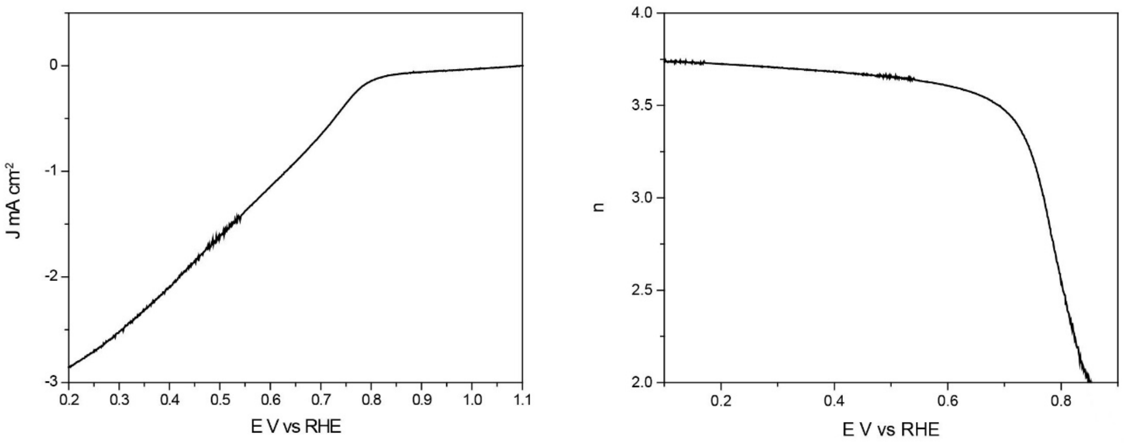


**Figure S17**. ORR LSV curve and charge transfer number of Co,N-codoped C nanowires. This shows that the ORR activity is poor with Co doping alone, and the ORR activity of the Pt-NC and PtCo-NC series is primarily due to Pt.

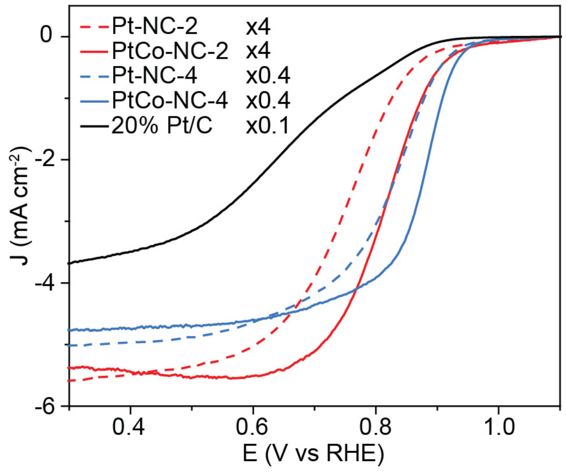


**Figure S18**. ORR polarization curves of the series of samples at the same Pt loading of ca. 3 $\mu g_{Pt}$ cm$^{-2}$. In comparison to the those in Figure 4a, the catalyst loading of Pt-NC-2 and PtCo-NC-2 was increased by a factor of 4 to 0.64 mg cm$^{-2}$; the loading of Pt-NC-4 and PtCo-NC-4 was reduced by 60% at 0.064 mg cm$^{-2}$; and the  loading of commercial 20% Pt/C was reduced by 90% to 0.016 mg cm$^{-2}$.

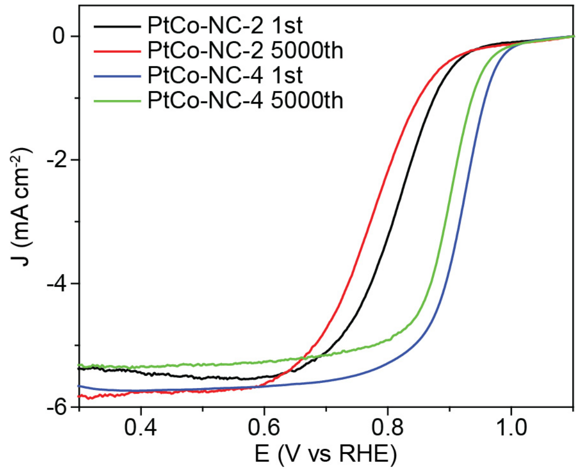


**Figure S19.** Stability tests of select samples. The catalyst loading of PtCo-NC-2 is 0.64 mg $cm^{-2}$ (or 3 $\mu g_{Pt}$ $cm^{-2}$), and that of PtCo-NC-4 is 0.16 mg $cm^{-2}$ (or 10 $\mu g_{Pt}$ $cm^{-2}$).

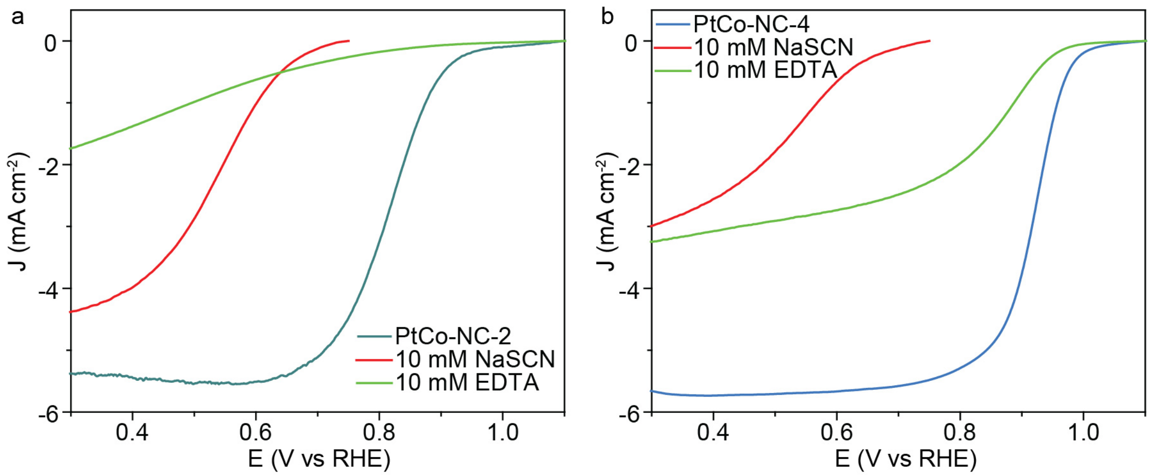


**Figure S20.** Poisoning test of (a) PtCo-NC-2 and (b) PtCo-NC-4. The NaSCN poisoning tests were carried out in 0.1 M $HClO_4$ with 10 mM NaSCN. The EDTA poisoning tests were measured in 0.1 M $HClO_4$ after the catalysts were treated in a solution of 1 M KOH and 10 mM EDTA at 60 °C overnight. The catalyst loading of PtCo-NC-2 was 0.64 mg $cm^{-2}$ (or 3 $\mu g_{Pt}$ $cm^{-2}$), and that of PtCo-NC-4 0.16 mg $cm^{-2}$ (or 10 $\mu g_{Pt}$ $cm^{-2}$).

**Table S1. Gibbs free energies of each step in ORR for a range of Pt-NC and PtCo-NC configurations as shown in Figure S1 at the potential of +0.9 V. The rate determined steps (RDS) are marked as red.**

| # of Pt+Co | # of Pt | configuration | $\Delta G_1$ (eV) | $\Delta G_2$ (eV) | $\Delta G_3$ (eV) | $\Delta G_4$ (eV) |
|---|---|---|---|---|---|---|
| 1 | 1 | Pt | 1.132 | 0.204 | -1.143 | -1.512 |
| 2 | 2 | PtPt | 1.10 | - | - | - |
| 2 | 1 | PtCo | 0.437 | -2.407 | 0.425 | 0.226 |
| 2 | 1 | PtCo* | 1.07 | - | - | - |
| 3 | 3 | $Pt_3$ | 0.619 | -0.729 | -0.498 | -0.712 |
| 3 | 1 | $Pt_1Co_2$ | -0.165 | -1.538 | 0.204 | 0.179 |
| 3 | 2 | $Pt_2Co_1$ | -0.316 | -1.571 | 0.185 | 0.393 |
| 4 | 4 | $Pt_4$ | 0.741 | - | - | - |
| 4 | 1 | $Pt_1Co_3$ | 0.638 | - | - | - |
| 4 | 2 | $Pt_2Co_2$ | -1.821 | -0.522 | 0.639 | 0.384 |
| 4 | 2 | $Pt_2Co_2$ | 0.909 | - | - | - |
| 4 | 2 | $Pt_2Co_2$* | -1.016 | -1.607 | 0.729 | 0.575 |
| 4 | 3 | $Pt_3Co_1$ | 0.703 | -1.603 | -0.403 | -0.016 |
| 6 | 6 | $Pt_6$ | -0.167 | -1.829 | 0.505 | 0.171 |
| 6 | 6 | $Pt_6$ | -0.033 | -1.975 | 0.517 | 0.171 |
| 6 | 1 | $Pt_1Co_5$ | 0.352 | -3.474 | 1.254 | 0.548 |
| 6 | 1 | $Pt_1Co_5$* | -0.69 | -2.18 | 1.209 | 0.345 |
| 6 | 3 | $Pt_3Co_3$ | 0.325 | -1.374 | -0.1 | -0.171 |
| 6 | 3 | $Pt_3Co_3$ | -0.585 | -1.87 | 0.479 | 0.655 |
| 6 | 3 | $Pt_3Co_3$ | -0.384 | -1.711 | 0.401 | 0.375 |
| 6 | 3 | $Pt_3Co_3$ | -0.031 | -1.248 | -0.158 | 0.118 |
| 6 | 5 | $Pt_5Co_1$ | -0.008 | -2.935 | 0.343 | 1.28 |
| 6 | 5 | $Pt_5Co_1$* | -1.152 | -0.111 | -0.077 | 0.021 |
| 9 | 9 | $Pt_9$ | 0.167 | -1.456 | 0.016 | -0.046 |
| 9 | 9 | $Pt_9$ | -0.187 | -1.062 | -0.128 | 0.057 |
| 9 | 1 | $Pt_1Co_8$ | 0.065 | -1.46 | 0.145 | -0.071 |
| 9 | 5 | $Pt_5Co_4$ | -1.786 | -1.534 | 2.1 | -0.1 |
| 9 | 8 | $Pt_8Co_1$ | -0.091 | -1.347 | 0.014 | 0.105 |
| 9 | 8 | $Pt_8Co_1$* | 0 | -1.224 | -0.14 | 0.044 |
| slab | slab | Pt | 0.139 | -0.71 | -0.535 | -0.215 |

**Table S2. Bader charges of selected structures in Figure S1.**

| | PtCo | PtCo* | Pt single atom | Co single atom | Pt1Co8 |
|---|---|---|---|---|---|
| Pt | 15.564 | 15.146 | 15.162 | | 16.72 |
| Co | 16.221 | 16.103 | | 16.054 | 16.3-16.6 |

**Table S3. Elemental analysis of the Pt-NC and PtCo-NC samples by ICP-OES measurements**

| sample | Pt %wt | sample | Pt wt% | Co wt% |
|---|---|---|---|---|
| **Pt-NC-1** | 0.25 | **PtCo-NC-1** | 0.25 | 0.5 |
| **Pt-NC-2** | 0.414 | **PtCo-NC-2** | 0.505 | 1.119 |
| **Pt-NC-3** | 1.391 | **PtCo-NC-3** | 0.954 | 2.638 |
| **Pt-NC-4** | 4.753 | **PtCo-NC-4** | 6.292 | 4.069 |

**Table S4. EXAFS fitting results**

| sample | element | bond | CN | R (Å) | $\sigma^2$ (Å) x$10^{-3}$ | $E_0$ (eV) | R factor |
|---|---|---|---|---|---|---|---|
| Pt-NC-1 | Pt | Pt-N | 3.5(9) | 1.99(2) | 7(4) | 9(3) | 0.0137 |
| Pt-NC-3 | Pt | Pt-N | 2.9(4) | 1.99(2) | 6(3) | 10(1) | 0.0105 |
| | | Pt-Pt | 2(1) | 2.71(3) | 3(5) | -1(5) | 0.0105 |
| PtCo-NC-1 | Pt | Pt-N | 3.6(3) | 2.001(9) | 7(1) | 9.4(8) | 0.0108 |
| | Co | Co-N | 8(3) | 2.03(4) | 17(7) | -6(4) | 0.0228 |
| | | Co-Pt | 3(1) | 2.81(3) | 7(5) | -6(4) | 0.0228 |
| PtCo-NC-3 | Pt | Pt-N | 1.7(8) | 1.95(5) | 7(3) | 7(4) | 0.0242 |
| | | Pt-Co | 3(2) | 2.65(2) | 7(3) | 7(4) | 0.0242 |
| | | Pt-Pt | 5(3) | 2.68(5) | 7(3) | 7(4) | 0.0242 |
| | Co | Co-N | 1.6(3) | 1.94(2) | 3(2) | -5(3) | 0.0242 |
| | | Co-Co | 1.8(4) | 2.50(2) | 3(2) | -5(3) | 0.0242 |
| | | Co-Pt | 1.8(4) | 2.65(2) | 3(2) | -5(3) | 0.0242 |

**Table S5. Fitting results of the Pt 4f XPS data**

| sample | Pt SA | | Pt-O | | Pt(0) | |
|---|---|---|---|---|---|---|
| | $4f_{7/2}$ | $4f_{5/2}$ | $4f_{7/2}$ | $4f_{5/2}$ | $4f_{7/2}$ | $4f_{5/2}$ |
| **Pt-NC-1** | 72.32 | 75.68 | 74.18 | 77.53 | | |
| **Pt-NC-2** | 72.52 | 75.86 | 73.91 | 77.26 | | |
| **Pt-NC-3** | 72.47 | 75.82 | 73.97 | 77.32 | | |
| **Pt-NC-4** | 72.38 | 75.73 | 74.2 | 77.55 | 71.37 | 74.73 |
| **PtCo-NC-1** | 72.27 | 75.63 | 73.86 | 77.21 | | |
| **PtCo-NC-2** | 72.36 | 75.71 | 73.89 | 77.24 | | |
| **PtCo-NC-3** | 72.27 | 75.62 | 73.93 | 77.28 | | |
| **PtCo-NC-4** | 72.37 | 75.72 | 74.19 | 77.54 | 71.57 | 74.92 |

**Table S6. Fitting results of the Co 2p XPS data**

| sample | Co SA | | satellite | | Co(0) | |
|---|---|---|---|---|---|---|
| | $2p_{3/2}$ | $2p_{1/2}$ | $2p_{3/2}$ | $2p_{1/2}$ | $2p_{3/2}$ | $2p_{1/2}$ |
| **PtCo-NC-1** | 780.68 | 796.19 | 785.79 | 801.29 | | |
| **PtCo-NC-2** | 780.79 | 796.29 | 785.9 | 801.40 | | |
| **PtCo-NC-3** | 780.66 | 796.16 | 786.13 | 801.63 | | |
| **PtCo-NC-4** | 780.64 | 796.14 | 785.47 | 803.23 | 778.36 | 793.41 |

**Table S7. Pt to Co atomic ratio by XPS measurements**

| sample | Pt:Co mass ratio | Pt:Co atomic ration |
|---|---|---|
| **PtCo-NC-1** | 5.07 | 1.53 |
| **PtCo-NC-2** | 4.33 | 1.31 |
| **PtCo-NC-3** | 5.65 | 1.70 |
| **PtCo-NC-4** | 5.58 | 1.69 |

**Table S8. Fitting results of the C 1s XPS data**

| sample | C $sp^2$ | C $sp^3$ | C-O |
|---|---|---|---|
| **Pt-NC-1** | 284.60 | 285.52 | 287.22 |
| **Pt-NC-2** | 284.72 | 285.51 | 287.84 |
| **Pt-NC-3** | 284.74 | 285.49 | 288.13 |
| **Pt-NC-4** | 284.76 | 285.6 | 287.89 |
| **PtCo-NC-1** | 284.67 | 285.53 | 287.74 |
| **PtCo-NC-2** | 284.72 | 285.47 | 287.90 |
| **PtCo-NC-3** | 284.67 | 285.51 | 288.03 |
| **PtCo-NC-4** | 284.68 | 285.5 | 287.94 |

**Table S9. Fitting results of the N 1s XPS data.**

| sample | Pyridinic (eV) | Pyrrolic (eV) | Graphitic (eV) | Oxidized (eV) |
|---|---|---|---|---|
| **Pt-NC-1** | 398.17 | 399.5 | 400.92 | 402.36 |
| **Pt-NC-2** | 398.19 | 399.36 | 401.07 | 402.84 |
| **Pt-NC-3** | 398.42 | 399.47 | 401.05 | 402.25 |
| **Pt-NC-4** | 398.39 | 399.53 | 401.08 | 402.14 |
| **PtCo-NC-1** | 398.37 | 399.79 | 401.03 | 402.30 |
| **PtCo-NC-2** | 398.49 | 399.84 | 401.06 | 402.25 |
| **PtCo-NC-3** | 398.56 | 399.87 | 401.04 | 402.57 |
| **PtCo-NC-4** | 398.57 | 399.85 | 401.06 | 402.73 |

**Table S10. Summary of the ORR activity**

| sample | $E_{onset}$ (V vs RHE) | $E_{1/2}$ (V vs RHE) | n at +0.7 V | $J_k$ at +0.85V (mA cm$^{-2}$) | $J_k$ at +0.85V (A mg$^{-1}$) | Tafel slope (mV dec$^{-1}$) |
|---|---|---|---|---|---|---|
| **Pt-NC-1** | 0.88 | 0.72 | 3.98 | 0.26 | 0.65 | 146.9 |
| **Pt-NC-2** | 0.93 | 0.74 | 3.96 | 0.43 | 0.63 | 121.4 |
| **Pt-NC-3** | 0.98 | 0.82 | 3.98 | 2.56 | 1.13 | 92.4 |
| **Pt-NC-4** | 1.01 | 0.87 | 4.00 | 5.9 | 0.76 | 73.5 |
| **PtCo-NC-1** | 0.94 | 0.77 | 3.98 | 0.61 | 1.5 | 106.3 |
| **PtCo-NC-2** | 0.96 | 0.78 | 3.98 | 0.71 | 0.88 | 107.4 |
| **PtCo-NC-3** | 1.00 | 0.87 | 3.99 | 6.43 | 4.16 | 77.4 |
| **PtCo-NC-4** | 1.03 | 0.93 | 3.96 | 19.75 | 1.93 | 53.7 |
| **20% Pt/C** | 0.99 | 0.86 | 3.92 | 2.83 | 0.09 | 79.5 |

**Note S1**

For a Pt single atom (Figure 1d), a high $\Delta G_{RDS}$ is observed at 1.13 eV, indicating unfavorable oxygen reduction activity. $\Delta G_{RDS}$ decreases slightly to 1.10 eV for dimeric $Pt_2$ (Figure S1). Yet, the addition of a third Pt atom to form trimeric $Pt_3$ leads to a marked decrease of $\Delta G_{RDS}$ to 0.62 eV at the central Pt atom. Further decrease of $\Delta G_{RDS}$ can be seen with a continuing increase of the Pt domain size; interestingly, $\Delta G_{RDS}$ of $Pt_9$ (ca. 0.9 nm in diameter, Figure 1f and Figure S1) diminishes to only 0.17 eV at the center and edge atoms, which is very comparable to that (0.14 eV) of Pt slab.

**Note S2**

Interestingly, incorporation of an adjacent Co atom forming a Pt-Co pair significantly enhances the ORR activity. For instance, for the simplest structure of a Pt-Co dimer ($Pt_1Co_1$, Figure 1e and Figure S1), $\Delta G_{RDS}$ is only 0.43 eV, markedly lower than those of monomeric $Pt_1$ (1.13 eV, Figure 1d) and dimeric $Pt_2$ (1.10 eV, Figure S1). For a linear trimer, $Pt_1Co_2$ (Figure S2) exhibits a low $\Delta G_{RDS}$ of 0.20 eV, as compared to $Pt_3$ (0.62 eV) and $Pt_2Co_1$ (0.39 eV). Notably, for an even larger cluster such as $Pt_1Co_8$ (Figure 1g and Figure S1), with Pt fully coordinated with Co, $\Delta G_{RDS}$ is as low as 0.14 eV, very comparable to that of a Pt slab.

Importantly, such activation can not only occur for Pt single atoms ($Pt_1Co_y$, $y \geq 1$), but also for larger Pt domains ($Pt_xCo_y$, $x \geq 2$ and $y \geq 1$). For example, $Pt_2Co_1$ (Figure S2) shows a $\Delta G_{RDS}$ of 0.39 eV, much lower than that of dimeric $Pt_2$ alone (1.10 eV). For larger clusters, one $Pt_3Co_3$ configuration shows a $\Delta G_{RDS}$ of only 0.12 eV, less than 1/5 of that (0.62 eV) of $Pt_3$; in the $Pt_5Co_y$ series, the central Pt atom of $Pt_5Co_1$* displays a $\Delta G_{RDS}$ of only 0.02 eV, drastically below those of $Pt_4$ (0.74 eV) and $Pt_6$ (0.51 eV); and in $Pt_8Co_y$, the $\Delta G_{RDS}$ for the center atom of $Pt_8Co_1$* is 0.05 eV, also significantly lower than those of $Pt_6$ (0.51 eV) and $Pt_9$ (0.15 eV). Among these various systems, the middle Pt sites (Figure S3) of $Pt_5Co_1$*, $Pt_1Co_8$ and $Pt_8Co_1$* show a remarkably low $\Delta G_{RDS}$ of 0.02 eV, 0.15 eV and 0.04 eV, very close to or even lower than that of the Pt slab. In these, the Co-Pt distance is a critical parameter. For example, when the Co-Pt distance is increased from 2.27 Å (direct Pt-Co bonding) to 4.01 Å (non-direct bonding) (Figure S5), $\Delta G_{RDS}$ of the Pt site increases accordingly from 0.43 to 1.07 eV, similar to that for $Pt_1$ alone (without Co, Figure 1d). These configurations can be found in Figure S1 and their activities are summarized in Table S1.

**Note S3**

Further structural insights were obtained in spectroscopic measurements. Figure S10 shows the XRD patterns of the two sets of samples. For samples prepared at relatively low metal loadings, only a broad peak centered at ca. 25° can be observed, due to the (002) diffraction of graphitic carbon, confirming successful carbonization of the resin precursors. The fact that no other diffraction peaks can be seen is also consistent with the absence of metal NPs in these samples. For Pt-NC-4, in addition to the carbon (002) diffraction, peaks at 40°, 46° and 67° can also be found, due to the (111), (200) and (220) diffractions of *fcc* Pt (card 00-001-1190).[*1*] Similar diffraction patterns can be found with PtCo-NC-4, except that the peak positions appear at slightly higher $2\theta$ angles, indicating lattice shrinkage due to alloying between Pt and Co in the NPs.[*2*] These observations are in good agreement with results from the TEM studies (Figure 2).

**Note S4**

The elemental compositions and valence states were then examined by XPS measurements. Figure S11 depicts the full spectra where the elements of C, N, and Pt can be clearly identified with the Pt-NC and PtCo-NC series (with Co for the latter). Figure S14a depicts the high-resolution scans of the Pt 4f electrons in the Pt-NC samples, where deconvolution yields two doublets. The first pair at ca. 72.5 eV and 75.8 eV can be ascribed to Pt atoms in Pt-N, whereas the other pair at 74.0 eV and 77.3 eV is indicative of Pt atoms in a higher oxidation state, as observed previously with Pt single atoms.[*3, 4*] For Pt-NC-1, Pt-NC-2, and Pt-NC-3 samples, no other peaks can be resolved, consistent with the absence of large particles. By contrast, the Pt-NC-4 sample displays an additional pair of peaks at 71.4 eV and 74.7 eV, due to metallic Pt in Pt NPs,[*5, 6*] as observed in TEM measurements (Figure 2). Similar characteristics are observed with the PtCo-NC series (Figure S14b), suggesting good comparability between the Pt-NC and PtCo-NC samples. Yet, it should be noticed that the Pt binding energy in the latter is ca. 0.2 eV lower than that in the former, signifying charge transfer from Co to Pt, in agreement with XANES and DFT results.

Consistent results are obtained in the corresponding Co 2p spectra (Figure S14c), which exhibit a pair of peaks at 780.7 eV and 796.2 eV, due to partial oxidation of the Co atoms. The Co(0) peak can only be found in the PtCo-NC-4 sample at 778.3 eV and 793.4 eV, due to the formation of PtCo alloy NPs. The other elements of C and N do not show any obvious difference among the samples (Figure S12 and 13). The fitting results are summarized in Table S5-9.

**Note S5**

In Raman measurements, similar D and G bands are clearly defined in all samples, further confirming successful carbonization of the resin precursors, and the fact that the D to G band intensity ratio is similar among the samples indicates a rather consistent carbon matrix (Figure S15).

**References**


[1] R. Jamil, M. Sohail, N. Baig *et al.*, Synthesis of Hollow Pt-Ni Nanoboxes for Highly Efficient Methanol Oxidation. *Scientific Reports,* vol. 9, pp. 15273, 2019.

[2] K. Jiang, D. Zhao, S. Guo *et al.*, Efficient oxygen reduction catalysis by subnanometer Pt alloy nanowires. *Science Advances,* vol. 3, no. 2, pp. e1601705, 2017.

[3] J. Liu, M. Jiao, L. Lu *et al.*, High performance platinum single atom electrocatalyst for oxygen reduction reaction. *Nature Communications,* vol. 8, no. 1, pp. 15938, 2017.

[4] J. Li, B. Zhang, Y. Chen *et al.*, Styrene hydrogenation performance of Pt nanoparticles with controlled size prepared by atomic layer deposition. *Catalysis Science & Technology,* vol. 5, no. 8, pp. 4218-4223, 2015.

[5] E. I. Vovk, A. V. Kalinkin, M. Y. Smirnov *et al.*, XPS Study of Stability and Reactivity of Oxidized Pt Nanoparticles Supported on TiO2. *The Journal of Physical Chemistry C,* vol. 121, no. 32, pp. 17297-17304, 2017.

[6] Q. R. Shi, C. Z. Zhu, M. H. Engelhard *et al.*, Highly uniform distribution of Pt nanoparticles on N-doped hollow carbon spheres with enhanced durability for oxygen reduction reaction. *Rsc Advances,* vol. 7, no. 11, pp. 6303-6308, 2017.
